# The cardiovascular outcomes, heart failure and kidney disease trials tell that the time to use Sodium Glucose Cotransporter 2 inhibitors is now

**DOI:** 10.1002/clc.23508

**Published:** 2020-11-09

**Authors:** Michael E. Johansen, Christos Argyropoulos

**Affiliations:** ^1^ Grant Family Medicine OhioHealth Columbus Ohio USA; ^2^ Division of Nephrology University of New Mexico School of Medicine Albuquerque New Mexico USA

**Keywords:** cardiovascular outcomes, chronic kidney disease, diabetes, heart failure, meta‐analysis, SGLT2 inhibitors

## Abstract

Sodium glucose contrasporter 2 inhibitors (SGLT2i) were initially introduced as a novel class of modestly effective antiglycemics. Over the last 5 years, multiple members of this class have been examined for their cardiovascular safety, effects on heart failure with reduced ejection fraction (HFrEF) and chronic kidney disease (CKD) in diverse populations with or without diabetes type 2. The plethora of studies and outcomes examined make it difficult for the practitioner to track the entirety of the evidence. SGLT2i improve cardiorenal outcomes and have a beneficial risk benefit ratio across populations with cardiovascular disease, HFrEF and kidney disease. In this quantitative review, we synthesize the data from the large outcomes trials about the benefits and risks of SGLT2i. SGLT2i reduce all cause, cardiovascular mortality, heart failure hospitalizations, need for dialysis and acute kidney injury as a *class effect* across a broad range of populations with diabetes Type 2 at risk for cardiovascular disease, patients with HFrEF or CKD with or without diabetes. While certain adverse events for example, diabetic ketoacidosis and genital mycotic infections are reproducibly increased by SGLT2i, the absolute increase in the risk of these complications is smaller than the absolute risk reductions conferred by SGLT2i. Other complications such as amputations, fractures and urinary tract infections are increased to a lesser degree, or not at all (e.g., hypoglycemia). Overall, SGLT2is appear to have a favorable safety profile and thus should be used by cardiologists, nephrologists, endocrinologists, primary care physicians when managing the cardiorenal risk of their patients.

## 1 INTRODUCTION

Sodium glucose co‐transporter 2 inhibitors (SGLT2i) do not exactly excel in A1c reduction since their antiglycemic effect is quite modest: a reduction in A1c of 0.81 to 1.02% in treatment naive patients and 0.57 to 0.63% on a metformin background.^1^ Even more disappointing from the perspective of A1c lowering is the dramatic loss of antiglycemic efficacy of these agents as kidney function declines.^2^, ^3^ Yet clinical trials in cardiovascular (safety) outcome trials (CVOT), trials in in heart failure with reduced ejection fraction (HFrEF) and in chronic kidney disease (CKD) have shown a dramatic improvement in cardiovascular, kidney and survival outcomes, across diverse populations of patients with and without diabetes type 2 (T2D).

The CVOTs of SGLT2s were conducted to comply with the Food and Drug Administration (FDA)'s 2008 mandate of that any new antidiabetic agents should be shown to be free of excess cardiovascular risk.^4^ The notion that antiglycemics may be associated with cardiovascular risk has been around since tolbutamide, a first generation sulfonylurea, was linked to higher cardiovascular mortality in the 1970s^5^. Nevertheless, the cardiovascular risk of *specific* antiglycemics remained underappreciated until the Phases 2 and 3 trials of the proliferator‐activated receptor agonists muraglitazar^6^ and rosiglitazone^7^, ^8^ showed increases in congestive heart failure, death and cardiovascular events. In combination with the 22% increased risk of all‐cause mortality in ACCORD trial,^9^ this data made it clear that the cardiovascular safety of antiglycemics should be proven in controlled trials.

The CVOTs reported to date include empagliflozin's EMPA‐REG Outcome,^10^ canagliflozin's integrated CANVAS Program^11^ (comprised of two trials: CANVAS and CANVAS‐R), dapagliflozin's DECLARE‐TIMI‐58^12^ and ertugliflozin's VERTIS‐CV.^13^ The EMPA‐REG OUTCOME trial,^10^, ^14^ demonstrated superiority of empagliflozin for the three‐point major cardiovascular event (MACE‐3: a composite of cardiovascular death, nonfatal myocardial infarction [MI] or nonfatal stroke), with significantly lower rates of cardiovascular death, hospitalization for heart failure (HHF), all cause death and kidney outcomes. Similar to empagliflozin, canagliflozin^11^ was demonstrated to be superior to placebo for the MACE‐3. On the other hand, dapagliflozin^12^ and ertugliflozin^13^ only achieved non‐inferiority in their respective cardiovascular safety trials, DECLARE‐TIMI‐58 and VERTIS‐CV.

DAPA‐HF^15^ and EMPEROR‐reduced^16^ recruited patients with or without T2D but with reduced ejection fraction (EF < 35%). Both studies showed highly significant reductions in the primary outcome of HHF or cardiovascular death irrespective of baseline kidney function, proteinuria, gender, race, diabetic status. CREDENCE^17^ and DAPA‐CKD^18^ examined the effects of canagliflozin and dapagliflozin in patients with persistent kidney damage, that is, persistent macroalbuminuria and impaired eGFR (between 30 and 90 ml/min/1.73 m^2^) on a background of maximally tolerated therapy with RASi. While CREDENCE recruited patients with type 2 diabetes, DAPA‐CKD also included patients without T2D. These CKD trials not only demonstrated that the drugs reduced a composite that involved worsening kidney function, the need for dialysis, and reduced cardiovascular outcomes.

The rapidly expanding landscape of the SGLT2i trials can be rather daunting to follow, given the variable study designs, populations, and the sequential testing strategy employed by the study authors. There remains understandable confusion about the relative merits of one SGLT2i versus another since individual study results tend to be presented as “significant” or “nonsignificant” without consideration of the totality of evidence across trials. In the present meta‐analysis we will synthesize the primary outcomes and the high‐profile adverse events, which to the authors' opinion seem to act as a barrier against the wider adoption of the SGLT2i.

## 2 MATERIALS AND METHODS

### 2.1 Eligibility criteria

We included all randomized, placebo‐controlled studies involving SGLT2i that included cardiovascular endpoints, such as cardiovascular (CV) death, MACE‐3 and its components, HHF, or composite renal end‐points of the four commercially available selective SGLT2i in the United States (canagliflozin, dapagliflozin, empagliflozin, ertugliflozin). Other prospective and nonrandomized studies were all excluded.

### 2.2 Search methods

We built an incremental version of the “renal outcomes” meta‐analysis,^19^ by translating the (OVID) search string of the authors to a PubMed version using the freely available tool Medline Transpose (https://medlinetranspose.github.io/). Using this query string (Appendix S1 Supplementary Material) we queried PubMed for studies appearing after 14 June 2019 (the end day of the previous meta‐analysis) until 22 October 2020.

### 2.3 Selection of studies

The two authors independently verified the abstract of the identified studies for meeting the inclusion and the exclusion criteria. For studies reported in multiple publications, the authors merged the baseline and follow up data from all publications. The Cochrane Risk of Bias tool^20^ was used to assess the potential for bias for each individual study analyzed.

### 2.4 Data abstraction and statistical analyses

Information about trial characteristics, primary and secondary outcomes efficacy measures (Hazard Ratios [HR] and their confidence intervals) were extracted from the source publications and converted to log‐HR and standard errors by standard formulas. For the analysis of adverse events, the number of events in the SGLT2i and placebo arms in each trial, were analyzed via a logistic regression and the log‐odds (log‐OR) ratio and standard error were extracted from the logistic model output. Log‐HRs and log‐ORs were synthesized via fixed (FE) and random effects (RE) meta‐analysis, to provide an overall Treatment Effect. The fixed effects model assumes that the treatment effect will be the same across all trials, while the random effects model assumes that the treatment effect in each of the trials, are similar but not identical. Statistical heterogeneity among the trials synthesized was quantified by the I^2^ statistic and the between study variance(τ^2^), which was calculated by REstricted Maximum Likelihood (REML). The *p*‐value of the Q test was used to test for the statistical significance of the observed heterogeneity. To explore replicability of these findings we calculated the prediction interval^21^: this is the 95% confidence interval for the treatment effects in a future trial. A prediction interval that is narrow and overlapping with the confidence interval of the treatment effect, suggests that future studies are unlikely to change the conclusion we can draw from the currently available trials. The analyses were conducted in Microsoft R open v3.5. Data and code used to generate the images for these meta‐analyses are included in the online software repository https://bitbucket.org/chrisarg/sglt2imetanalysis.

## 3 RESULTS

The initial quantitative synthesis^19^ had screened 2085 papers to include four main trial publications, eight secondary analysis papers, and one correspondence letter for three cardiovascular safety trials EMPA‐REG Outcome, CANVAS Program, DECLARE‐TIMI‐58 and one kidney outcomes trial, CREDENCE. Out of these scientific publications eight included data relevant to the aims of our meta‐analysis and considered further. The Consort diagram for the updated meta‐analyses is shown in Supplementary Figure 1. Since mid‐2019 there had been an additional 109 papers (Citations in Supplement) fulfilling our search criteria, however only an additional study reports met our inclusion criteria: a cardiovascular safety trial (VERTIS‐CV), two heart failure trials (DAPA‐HF and EMPEROR‐Reduced) and an additional kidney outcomes trial (DAPA‐CKD). The risk of bias was low for all trials and all indicators (Supplementary Table 1). Our final dataset consisted of eight randomized controlled trials of 59 747 patients: 4 were CVOT safety trials, two targeted individuals with HFrEF, and two were kidney outcomes trials in patients with type 2 diabetes (Table 1). On average these studies followed participants for a median of 1.5–4.2 years; most participants (>65%) were men and of middle age (average age 63.1–66.8). All participants had type 2 diabetes except for three trials that recruited individuals with HFrEF and the kidney outcome trial DAPA‐CKD. Even though these trials recruited throughout the globe most participants were white (53.2% – 87.8%). Inhibitors of the Renin Angiotensin System (RASi) were used by the majority of the study participants (~80% in the trials not requiring such an inhibitor at baseline and > 98% in the kidney outcomes trials thar required participants to be on maximally tolerated RASi).

**TABLE 1:** Baseline characteristic of randomized controlled trials of the commercially available SGLT2i in the United States

	Empagliflozin		Canagliflozin		Dapagliflozin			Ertugliflozin
	EMPA‐REG Outcome^10^, ^14^, ^31^	EMPEROR ‐ REDUCED^16^	CANVAS Program^11^	CREDENCE^17^, ^32^	DECLARE‐TIMI 58^12^, ^33^	DAPA‐HF^15^	DAPA‐CKD^18^	VERTIS‐CV^13^
Primary Outcome	MACE‐3	CV Death/HHF	MACE‐3	WKD/ESKD/CV Death/Renal Death	MACE‐3	WHF / CV Death	WKD/ESKD/ CV Death/ Renal Death	MACE‐3
N of participants	7020	3730	10 142	4401	17 160	4744	4304	8246
Median follow up (y)	3.1	1.3	2.4	2.6	4.2	1.52	2.4	3.0
Region								
Europe	2885 (41.1)	1353 (36.3)	NR	864 (19.6)	7629 (44.5)	2154 (45.4)	1233 (28.6)	4637 (56.2)
North America	1394 (19.9)	425 (11.4)	NR	1182 (26.9)	5468 (31.9)	677 (14.3)	813 (18.9)	1813 (22)
Asia	1347 (19.2)	493 (13.2)	NR	NR	2186 (12.7)	1096 (23.1)	1346 (31.3)	523 (6.3)
Latin America	1081 (15.4)	1286 (34.5)	NR	941 (21.4)	1877 (10.9)	817 (17.2)	912 (21.2)	723 (8.8)
Rest of the world	313 (4.5)	173 (4.6)^a^	NR	1414 (32.1)	Νονε	None	None	550 (6.7)^b^
Women	2004 (28.5)	3730 (23.9)	3633 (35.8)	1494 (33.9)	6422 (37.4)	1109 (23.4)	1425 (33.1)	2477 (30.0)
Age (y)	63.1 ± 8.6	66.8 ± 11	63.3 ± 8.3	63.0 ± 9.2	63.9 ± 6.8	66.3 ± 10.9	61.9 ± 12.1	64.4 ± 8.1
Race/ethnicity								
White	5081 (72.4)	2629 (70.5)	7944 (78.3)	2931 (79.6)	13 653 (79.6)	3333 (70.3)	2290 (53.2)	7240 (87.8)
Asian	1517 (21.3)	672 (18)	1284 (12.7)	877 (19.9)	2303 (13.4)	1116 (23.5)	1467 (34.1)	498 (6.0)
Black	357 (5.1)	257 (6.9)	336 (3.3)	224 (5.1)	603 (3.5)	226 (4.8)	191 (4.4)	235 (2.8)
Other/NA	65 (0.9)	172 (4.6)	587 (5.7)	369 (8.4)	601 (3.5)	69 (1.5)	356 (8.3)	273 (3.3)
Diabetes (%)	100%	49.8%	100%	100%	100%	41.8%	67.7%	100%
Hb A1c	8.1 ± 0.8	NR	8.2 ± 0.9	8.3 ± 1.3	8.3 ± 1.2	NR	NR	8.2 ± 1.0
Duration of diabetes	57% > 10 y	NR	13.5 ± 7.8	15.7 ± 8.7	11.9 ± 7.8	NR	NR	13.0 ± 8.3
Cardiac/cardiovascular diseases								
Coronary artery disease	5308 (75.6)	1929 (51.7)^c^	5721 (56.4)	1313 (29.8)	5648 (32.9)	2674 (56.4)^c^	1710 (39.7)^d^	6256 (75.9)
Cerebrovascular disease	1637 (23.3)	NR	1958 (19.3)	700 (15.9)	1301 (7.6)	NR		1889 (22.9)
Peripheral arterial disease	1461 (20.8)	NR	7324 (72.2)	47.5 (1.1)	1025 (6)	NR		1541 (18.7)
History of heart failure	706 (10.1)	3730 (100)	1461 (14.4)	652 (14.8)	1724 (10)	4744 (100)	468 (10.9)	1958 (23.7)
SBP (mmHg)	135.4 ± 17	122 ± 15.7	136.6 ± 15.8	140 ± 15.6	135 ± 15.4	121.8 ± 16.3	137.1 ± 17.4	133.4 ± 13.8
DBP (mmHg)	76.7 ± 9.9	NR	77.7 ± 9.6	78.3 ± 9.4	78 ± 9.1	NR	77.5 ± 10.5	76.7 ± 8.4
LDL (mg/dl)	85.6 ± 35.7	NR	90.6 ± 34.8	96.5 ± 41.3	87.6 ± 35.4	NR	NR	89.1 ± 38.2
HDL (mg/dl)	44.4 ± 11.7	NR	46.4 ± 11.6	44.5 ± 13.5	47.2 ± 13	NR	NR	43.8 ± 12.1
Triglycerides (mg/dl)	170.6 ± 126.9	NR	177.1 ± 123	197.9 ± 144.3	178.2 ± 134.4	NR	NR	180.6 ± 114.6
BMI (kgr/m2)	30.6 ± 5.2	27.9 ± 5.4	31.9 ± 5.9	31.3 ± 6.2	32.1 ± 6.1	28.2 ± 5.9	29.5 ± 6.2	31.9 ± 5.4
Renal status								
eGFR	74 ± 21.4	62 ± 21.6	76.5 ± 20.5	56.2 ± 18.2	85.3 ± 15.9	65.8 ± 19.5	43.1 ± 12.3	76 ± 20.9
eGFR ≥90	1538 (21.9)^e^	NR	2476 (24.4)	211 (4.8)	8162 (47.6)	8162 (47.6)	None	NR
eGFR 60–90	3661 (52.2)^e^	NR	5625 (55.5)	1558 (35.4)	7732 (45.1)	7732 (45.1)	454 (10.5)	NR
eGFR <60	1819 (25.9)^e^	906 (12.9)	2039 (20.1)	2631 (59.8)	1265 (7.4)	1265 (7.4)	3850 (89.5)	1807 (21.9)
Mild albuminuria	4171 (60.0)^e^	NR	7007 (69.1)	31 (0.7)	11 644 (69.1)	11 644 (69.1)	NR	NR
Moderate albuminuria	2013 (29.0)^e^	NR	2266 (22.3)	496 (11.3)	4029 (23.9)	4029 (23.9)	NR	NR
Severe albuminuria	769 (11.1)^e^	NR	760 (7.5)	3874 (88)	1169 (6.9)	1169 (6.9)	2079 (48.3)	NR
Medications								
RASi	5712 (81.4)	2600 (69.7)	8116 (80)	4395 (99.9)	13 950 (81.3)	3968 (83.6)	4224 (98.1)	6686 (81.1)
Beta‐blockers	4554 (64.9)	3533 (94.7)	5421 (53.5)	1770 (40.2)	9030 (52.6)	4558 (96.1)	NR	5692 (69)
Antiplatelet agents^f^	6293 (89.6)	NR	7466 (73.6)	2624 (59.6)	10 487 (61.1)	NR	NR	6978 (84.6)
Statins	5403 (77)	NR	7599 (74.9)	3036 (69)	12 868 (75)	2794 (58.9)	2794 (64.9)	6747 (81.8)
MRA	441 (6.3)	2661 (71.3)	NR	NR	NR	3370 (71)	NR	674 (8.2)
Diuretics	3035 (43.2)	NR	4490 (44.3)	2057 (46.7)	6967 (40.6)	4433 (93.4)	1882 (43.7)	3542 (43)
ARNI	NR	727 (19.5)	NR	NR	NR	508 (10.7)	NR	NR
Insulin	3387 (48.2)	NR	5095 (50.2)	2884 (65.5)	7013 (40.9)	540 (11.4)	NR	3900 (47.3)
Metformin	5193 (74.0)	NR	7825 (77.2)	2545 (57.8)	14 068 (82)	1016 (21.4)	NR	6292 (76.3)
Sulfonylureas	3006 (42.8)	NR	4361 (43)	1268 (28.8)	7322 (42.7)	438 (9.2)	NR	3390 (41.1)
DPP4i	796 (11.3)	NR	1261 (12.4)	751 (17.1)	2888 (16.8)	310 (6.5)	NR	911 (11)
GLP1‐RA	196 (2.8)	NR	407 (4)	183 (4.2)	750 (4.4)	21 (0.4)	NR	278 (3.4)

*Note*: Unless stated otherwise, summaries are reported as N (%) or mean ± SD. Albuminuria is graded according to KDIGO as mild (UACR <30 mg/g), moderate (UACR: 30‐300 mg/g) and severe (UACR >300 mg/g). DAPA‐CKD graded albuminuria as non‐nephrotic (UACR <1000 mg/g) or nephrotic (UACR >1000 mg/g).

Abbreviations: ARNI, angiotensin receptor‐neprilysin inhibitor; BMI, body mass index; CV death/HHF, cardiovascular death or hospitalization for heart failure; DBP, diastolic blood pressure; DPP4i, dipeptidyl peptidase‐4 inhibitor; eGFR, estimated glomerular filtration rate (ml/min/1.73 m^2^); GLP1‐RA, glucagon‐like peptide‐1; MACE‐3, major adverse cardiovascular events (cardiovascular death; non fatal myocardial infarction or stroke); MRA, mineralocorticoid receptor antagonist; NR, not reported; RASi, renin angiotensin system inhibitor; either an angiotensin converting enzyme inhibitor or an angiotensin receptor blocker; SBP, systolic blood pressure; UACR, urinary albumin to creatinine ratio; WHF/CV Death, worsening heart failure (hospitalization or urgent intravenous therapy) or cardiovascular death; WKD/ESKD/CVD/renal death, worsening kidney function (reduction of eGFR by 50%) or end stage kidney disease or cardiovascular death or renal death.

^a^ India and Australia were included in this category.

^b^ Australia, New Zealand and Africa.

^c^ In this analysis coronary artery disease was inferred if the cause of heart failure was ischemic cardiomyopathy.

^d^ Primary publication reported only a grand total of cardiovascular disease which included the components of coronary artery disease, peripheral arterial disease, stroke, atrial and ventricular arrhythmias.

^e^ Percentages adjusted for missing baseline data.

^f^ Antiplatelet agents may include aspirin, clopidogrel.

**FIGURE 1:**
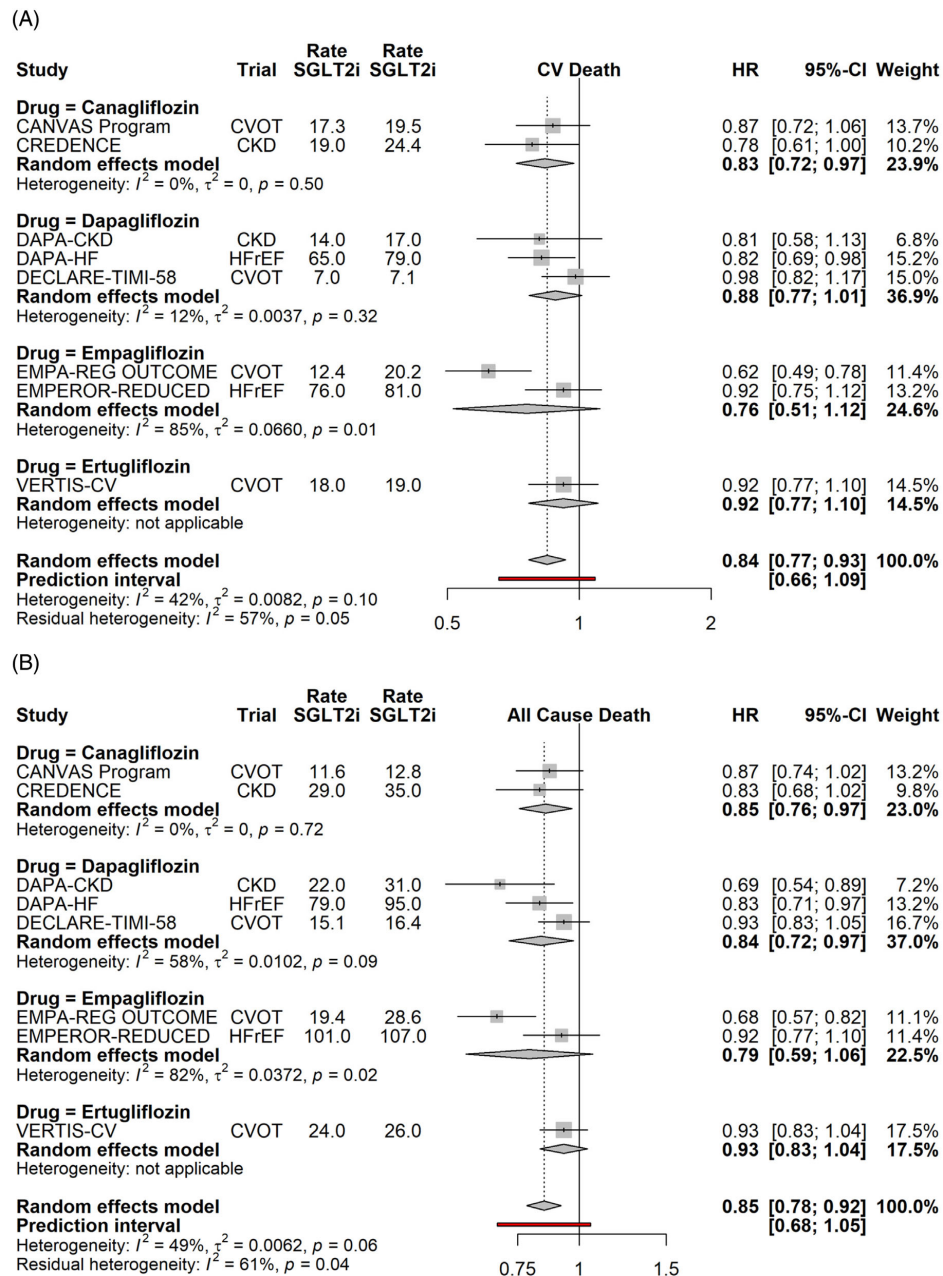
Effects of SGLT2i on cardiovascular (a) and all cause death (b), by drug. Random effects model synthesizes the effect across all studies, and the prediction interval gives the 95% range for the result of a future SGLT2i trial. Event rates (per 1000 patient years) are shown for both the SGLT2i and the placebo arms. CKD, chronic kidney disease; CVOT, cardiovascular outcome trial; HFrEF, heart failure with reduced ejection fraction; HR, hazard ratio

SGLT2i reduce cardiovascular (Figure 1(A)) and all‐cause mortality (Figure 1(B)) by 16% (*p* = .0006) and 15% (*p* < .0001) across populations of high cardiovascular risk as a result of diabetes Type 2, HFrEF and CKD (with or without diabetes) with little evidence of heterogeneity by drug (*p* value for the Q test 0.10 and 0.07 for cardiovascular and all cause death). One may be tempted to assign the suggestion of heterogeneity to the numerically smaller risk reduction seen in the VERTIS‐CV trial, but it Is in line with the heterogeneity seen in both empagliflozin (for all cause and cardiovascular death) and dapagliflozin (for all cause death). For example: the risk of death was reduced to a greater degree in the CVOT of empagliflozin compared to its HFrEF trial, but this pattern was reversed with dapagliflozin.

**FIGURE 2:**
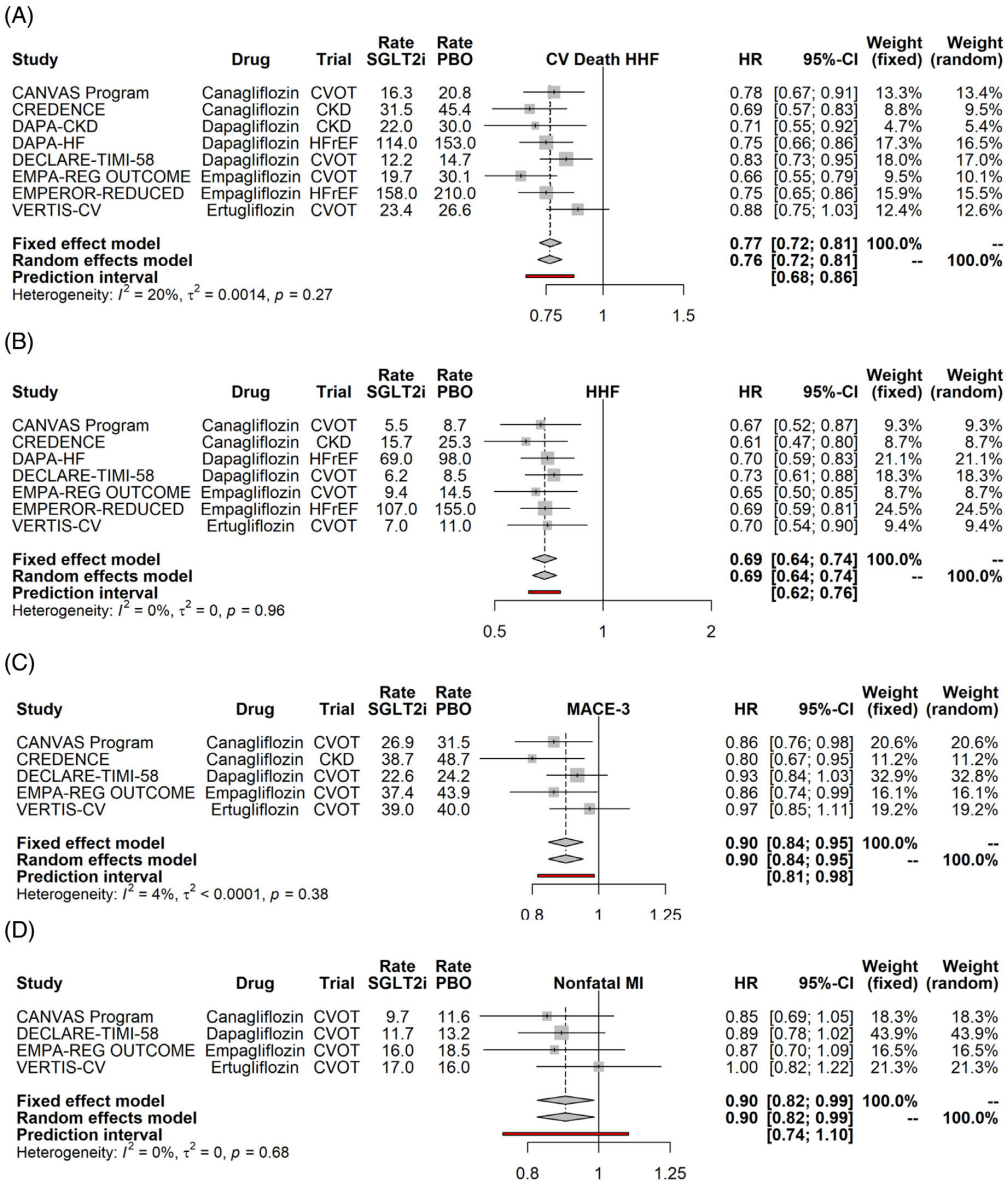
Effects of SGLT2i on the composite outcome of cardiovascular death or heart failure hospitalization (a) hospitalization for heart failure (b), the three point major adverse cardiovascular events (c) and nonfatal myocardial infarction (d). Random effects model synthesizes the effect across all studies, and the prediction interval gives the 95% range for the result of a future SGLT2i trial. Event rates (per 1000 patient years) are shown for both the SGLT2i and the placebo arms. CKD, chronic kidney disease; CVOT, cardiovascular outcome trial; HFrEF, heart failure with reduced ejection fraction; HHF, hospitalization for heart failure; HR, hazard ratio

Similarly, SGLT2i reduced the composite of HHF or cardiovascular death and HHF (Figure 2(A) and (B)), by 31% and 24% (*p* < .0001 for both) without any evidence of heterogeneity by study drug (Supplementary Figure 1).

We also examined the effects of SGLT2i on MACE‐3 (Figure 2(C)), Nonfatal MI (Figure 2(D)), Nonfatal stroke (Supplementary Figure 2) and the composite kidney outcome of worsening kidney function (which trials variably defined as >40% drop in the eGFR, doubling of the serum creatinine) or end stage kidney disease (ESKD), in need of renal replacement (Supplementary Figure 3). None of these outcomes showed any evidence for heterogeneity, so the effects of SGLT2i were examined as a class, rather than by drug. Whereas SGLT2i reduced MACE‐3 by 10% (*p* value of FE and RE were both .0002) and nonfatal MI by a similar amount (FE and RE *p* value were both .024), they had no effect on stroke. SGLT2i reduced worsening kidney function or ESKD by 39% (*p* < .0001). The beneficial effect on MACE‐3/MI and kidney outcomes were observed irrespective of the study drug, and trial type. Examination of the prediction intervals, shows that the effects on MACE‐3 and kidney function are very likely to be recapitulated in future SGLT2i trials, while some uncertainty exists about the replication of the effect on MI.

**FIGURE 3:**
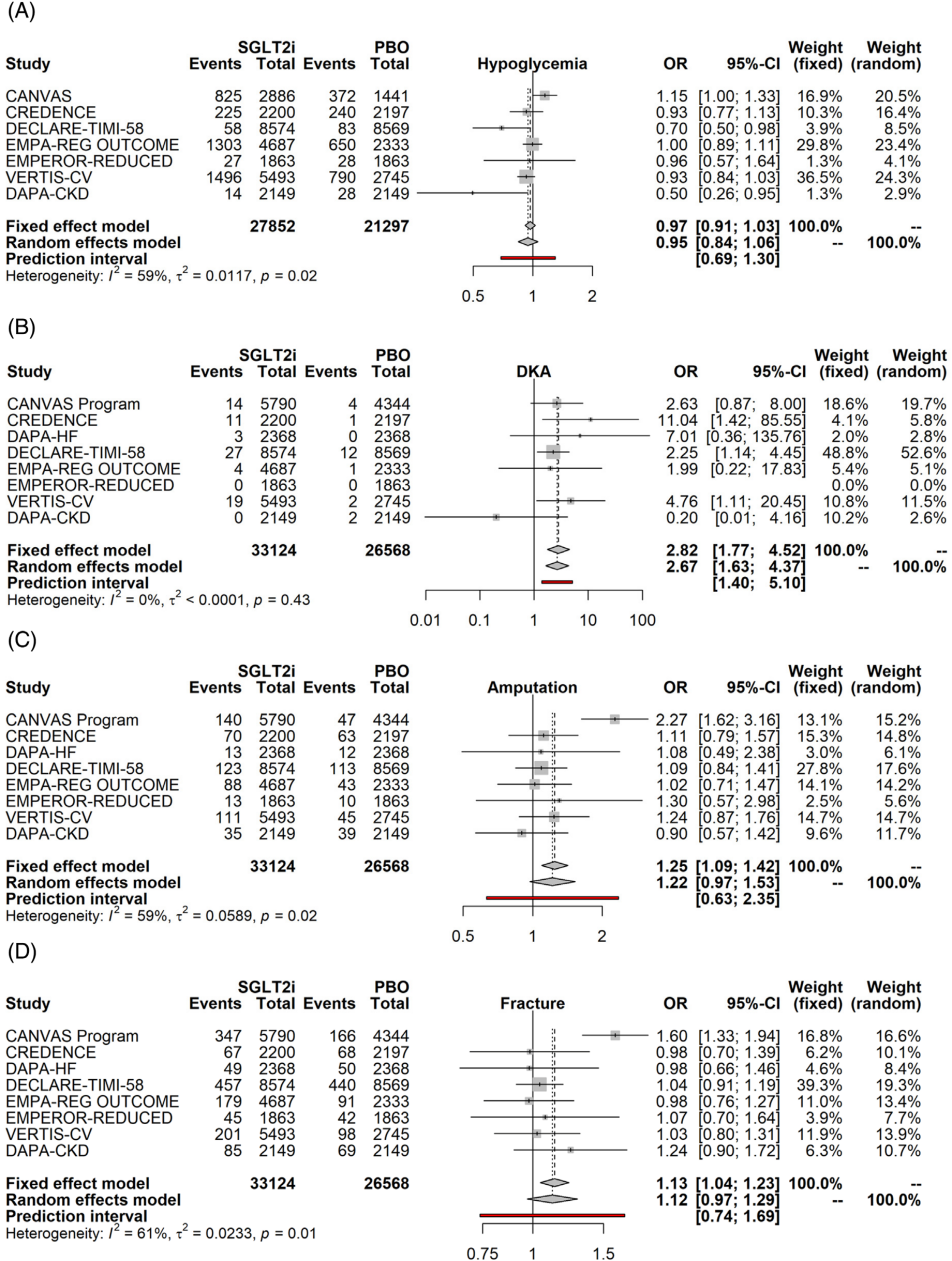
SGLT2i and adverse events: hypoglycemia (a) diabetic ketoacidosis (b), amputation (c) and fracture (d). Fixed and Random effects model synthesize the odds ratio across all studies, and the prediction interval gives the 95% range for the result of a future SGLT2i trial. Side effects for canagliflozin were available either from the CANVAS trial or the integrated dataset of the CANVAS/CANVAS‐R trials (CANVAS Program) as event rates; they were converted to events by multiplying the event rate and the sample size in each arm of the study. CKD, chronic kidney disease; CVOT, cardiovascular outcome trial; DKA, diabetic ketoacidosis; HFrEF, heart failure with reduced ejection fraction; HHF, hospitalization for heart failure; OR, odds ratio

SGLT2i are unlikely to cause hypoglycemia (Figure 3(A), OR of 0.95, *p* = .35), but are associated with reproducible increases in the rate of diabetic ketoacidosis (Figure 3(B), DKA, OR 2.87, *p* < .001). Whereas the rate of amputations appears to be increased in participants receiving SGLT2i, the magnitude of the effect appears to be small (OR 1.22), the statistical significance of the association dependent on the statistical model used (Figure 3(C), FE *p* = .0012, RE *p* = .0892), and considerable uncertainty remains about the observed effect in future trials. Similarly, SGLT2i are associated with small increase in fractures, that is, OR of 1.13 (Figure 3(D), FE *p* = .0029, RE *p* = .1283) and considerable uncertainty of the rate in future trials.

**FIGURE 4:**
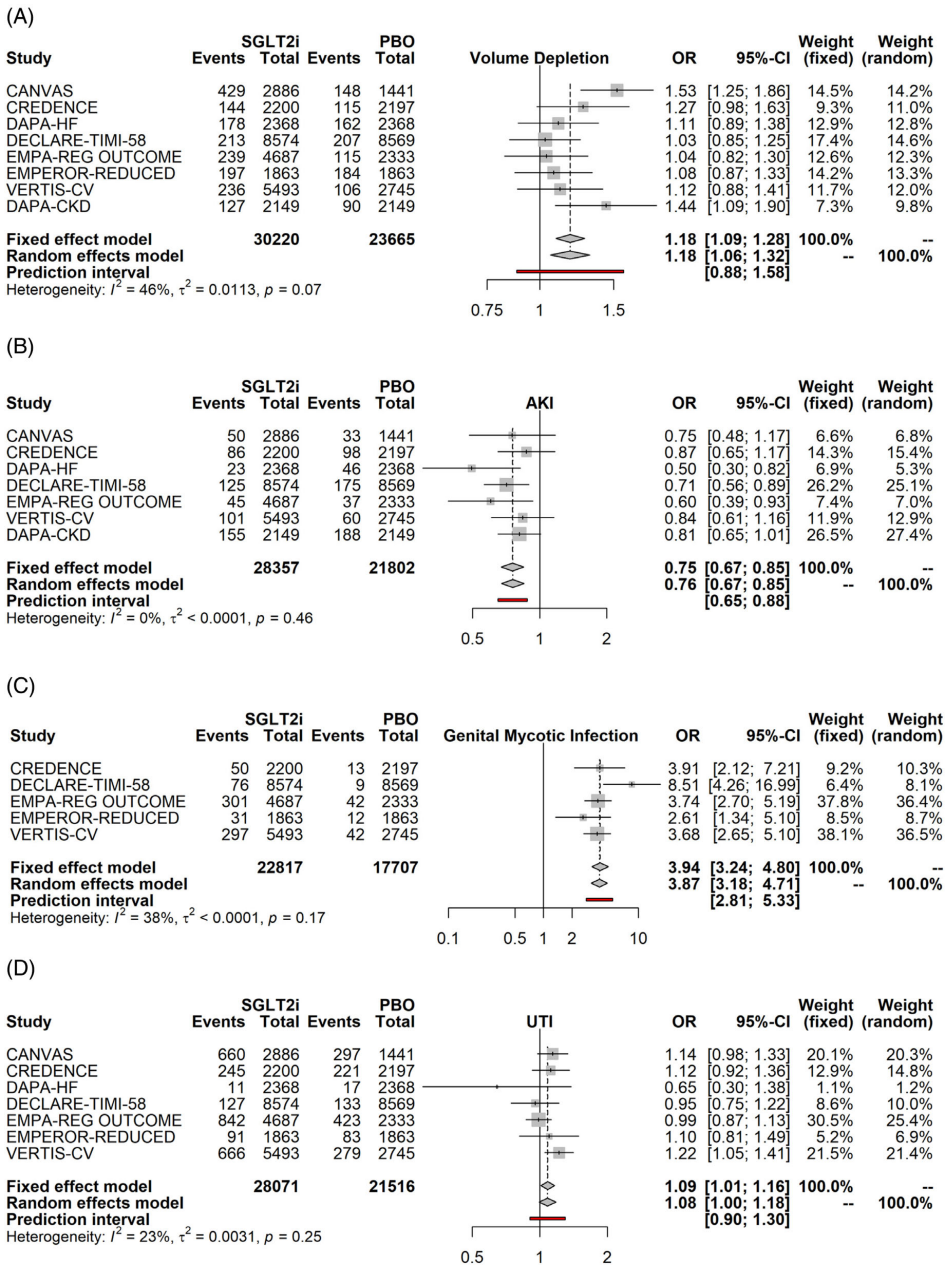
SGLT2i and renal/infectious adverse events: volume depletion (a) acute kidney injury (b), genital mycotic infections (c) and urinary tract infection (d). Fixed and random effects model synthesize the odds ratio across all studies, and the prediction interval gives the 95% range for the result of a future SGLT2i trial. Side effects for canagliflozin were available either from the CANVAS trial or the integrated dataset of the CANVAS/CANVAS‐R trials (CANVAS Program). AKI, acute kidney injury; CKD, chronic kidney disease; CVOT, cardiovascular outcome trial; DKA, diabetic ketoacidosis; HFrEF, heart failure with reduced ejection fraction; HHF, hospitalization for heart failure;OR, odds ratio; UTI, urinary tract infection

Volume depletion was increased to a small extent by SGLT2i (Figure 4(A), OR: 1.18, FE *p* < .001, RE *p* = .0026), but acute kidney injury (AKI) was *reduced* by 25% (Figure 4(B), *p* < .001), with the latter effect expected to be highly reproducible given the narrow width of the prediction interval. Mycotic infections were increased to a large extent by SGLT2i (Figure 4(C), OR of 3.9, *p* < .0001 for both FE and RE), but urinary tract infections (UTI) to small degree (Figure 4(D), OR 1.08, FE *p* = .0217, RE *p* = .0651).

## 4 DISCUSSION

The coming of age of SGLT2i, that started with the publication of the EMPA‐REG Outcome in 2015, came full circle in the summer of 2020 with the completion of DAPA‐CKD: what started off as a rather un‐exciting class of antiglycemics, has unequivocally shown broad (cardio)renal benefits in patients with diabetes at risk for cardiovascular disease, cardiovascular benefit in patients with diabetic or non‐diabetic kidney disease, and heart failure benefit in patients with known HFrEF or CKD with or without diabetes. In this meta‐analysis we provide a quantitative summary of the effects of SGLT2i, by integrating the efficacy and safety findings from the large outcome trials.

SGLT2i reduce all cause and cardiovascular mortality, the 3 point MACE, HHF, MI, while also stabilizing kidney function, reducing the risk of dialysis and the odds of AKI in a dose independent manner. Out of these findings, the ones most likely to replicate in future clinical trials are the MACE‐3, HHF and kidney specific effects. The current evidence points to a neutral effect on stroke. The price to pay for these benefits are higher rates of genital infections and DKA, and to a much smaller and less reproducible extent, UTIs, fractures and amputations. Benefits and harms are consistently observed across trial populations, with little if any evidence of heterogeneity by study drug, i.e. these appear consistent with class rather than drug specific effects. Similar to anti‐hypertensive in‐class differences, the challenge in gaining stronger evidence of in‐class differences is the extremely large sample size that would be required for the expected small differences.

While the benefits of SGLT2i are large, clinically meaningful, and robust, they come with certain side effects that appear to be rather unpalatable e.g. mycotic infections, or serious, disfiguring, or even potential lethal e.g. amputations, fractures or DKA. Providers may thus hesitate to prescribe SGLT2i, and patients resistant to take them. However, any apprehension about SGLT2is disappears once the frequency of these side effects is contrasted to the benefits. This is best understood via specific examples. In EMPEROR‐Reduced, 24.7% versus 19.3% individuals experienced the primary outcome of HHF or cardiovascular death in the placebo and SGLT2i arms. On the other hand, the impressive OR of 3.9 for mycotic infections translates to an absolute increase in the probability of this event from 0.6% to 1.7%. A patient or a provider may value one death/HHF event averted, to be almost five times (since [24.7–19.3]/[1.7–0.6]~5) less valuable than one additional mycotic infection for them not to use SGLT2i. Similarly, the absolute increase in the rate of diabetic ketoacidosis and amputations in the canagliflozin arm of CREDENCE over placebo was 2.0 and 1.1 episodes per 1000 patient years. However, use of canagliflozin also resulted in absolute rate reductions of cardiovascular mortality and ESKD by 5.4 and 13.4 events per 1000 patient years, while reducing the rate of HHF by 9.6 events per 1000 patient years. For most patients, the small absolute increase in side effects would represent a highly acceptable trade‐off to avoid (cardiovascular) death or dialysis.

Several meta‐analyses about the effects of SGLT2i were published in late 2019 and early 2020 ^19^, ^22^, ^23^, ^24^, ^25^, ^26^, ^27^ with different focus (e.g., cardiovascular or renal outcomes or safety) and different search strategies than our own. The strengths of the present meta‐analysis are the simultaneous consideration of a broad range of both efficacy AND safety outcomes, the inclusion of four large studies and our focus on an expanded population of patients of extremely high cardiorenal risk. We addressed the heterogeneity in the population by resorting to random effects approaches, as it is likely for the effects of SGLT2is to differ among populations recruited in the trials. The main limitation of our meta‐analysis is that it omitted smaller studies from the phase 3 programs that led to the introduction of SGLT2i as antiglycemics and from those SGLT2i not currently licensed in North America, for which outcomes studies are still lacking. We addressed this limitation by reporting prediction intervals, that is, plausible ranges of hazard ratios that may be observed in future trials. This statistical computation, which is not commonly reported in meta‐analyses, provides a snapshot of outcomes for which some uncertainty remains (e.g., all cause mortality) and others (e.g., HHF or kidney outcomes) for which minimal residual uncertainty exists about future attempts to replicate these findings.

Given the favorable risk benefit ratio, how do we get SGLT2i to be used more widely? We hope that by synthesizing the data, our meta‐analysis will inform the practice of the “four” key specialties (cardiologists, nephrologists, endocrinologists and primary care). When using these drugs, a few commonsense rules should be employed to reduce risk of adverse events. Providers should consider temporarily discontinuing SGLT2i in clinical situations that predispose to ketoacidosis, for example, prolonged fasting, post‐surgery or an acute illness, a strategy known as the “sick day rule.” Peripheral arterial disease (PAD) is a major risk factor for atherosclerotic cardiovascular disease, and nearly 20%–25% of participants in these trials had PAD. Given the results of CANVAS, there has been concern about using SGLT2i in individuals with PAD. However, withholding SGLT2i from patients with PAD does not seem wise given the known benefits and the relative risk of amputations in other trials (including canagliflozin's CREDENCE). Such considerations led the FDA to remove the black box warning of amputations in August 2020. Nevertheless, it is reasonable to avoid SGLT2i in patients with active PAD (e.g. critical ischemia, non‐healing ulcers as was done in CREDENCE) and discuss the potential for this complication in all other patients initiated on SGLT2i, while following them clinically for signs of incident or worsening PAD. Understanding the effects of these drugs on kidney function would also seem important; nearly all SGLT2i still carry warnings about AKI because of a major misunderstanding about the effects of these drugs on the eGFR during their launch as antiglycemics. All SGLT2i will cause an immediate and reversible 4–6 ml/min/1.73m^2^ drop in eGFR after initiation^17^, ^18^ and thus kidney function should be checked within 3–4 weeks to establish the patient's new eGFR baseline. Additionally, the combined use of SGLT2i with RASi is not only nephroprotective as shown in CREDENCE, DAPA‐CKD and subgroup analyses of CVOTs, but is associated with *reduced* rates of AKI, an effect seen in both the large trials and multiple observational cohorts.^28^, ^29^ Attention to volume status, may prevent the few AKIs due to volume depletion that are occasionally observed. Finally the incidence of genital infections may be reduced nearly 10 fold by simple personal hygiene advice that involves washing the urogenital area with water after each void and before going to bed.^30^

Armed with the knowledge of benefits, risks, and some clinical sense about risk reduction, the four key specialties should no longer hesitate to discuss SGLT2i for cardiorenal protection with their patients.

## 5 CONCLUSIONS

SGLT2i reduce all cause, cardiovascular mortality, heart failure hospitalizations, need for dialysis and acute kidney injury as a *class effect* across a broad range of populations with diabetes Type 2 at risk for cardiovascular disease, patients with HFrEF or CKD with or without diabetes. Overall, SGLT2is appear to have a favorable safety profile and thus should be used by the four “key specialties” (cardiology, nephrology, endocrinology, primary care) first and foremost as agents that reduce cardiorenal and heart failure risk and only secondarily as antiglycemics.

## CONFLICT OF INTEREST

Michael E Johansen has nothing to disclose. Christos Argyropoulos has received consulting fees from Bayer, Baxter Healthcare, Health Services Advisory Group and research support from Dialysis Clinic, Inc.

## Supporting information


**Appendix S1**: Supporting Information

## Data Availability

The data and software code that support the findings of this study are openly available at repository https://bitbucket.org/chrisarg/sglt2imetanalysis.
