# Supplementary material for: The cardiovascular outcomes, heart failure and kidney disease trials tell that the time to use Sodium Glucose Cotransporter 2 inhibitors is now
Source: Clin Cardiol. 2020 Nov 9;43(12):1376–87. doi: 10.1002/clc.23508 (PMC7724239; doi:10.1002/clc.23508)
Supplement: Supplementary file 1 — Appendix S1: Supporting Information [file CLC-43-1376-s001.docx]

**Search String Used in the Meta-analysis**

(Sodium-Glucose Co-Transporter*[tiab] OR "Sodium-Glucose Transporter 2 Inhibitors"[mesh] OR "dapagliflozin*"[All Fields] OR "canagliflozin*"[All Fields] OR "empagliflozin*"[All Fields] OR "ertugliflozin*"[All Fields]) AND 2019/06/14:2020/10/30[Date - Publication] AND (("diabetes mellitus, type 2"[MeSH Terms] OR "diabetes mellitus, type 2/drug therapy"[MeSH Terms]) AND 2019/06/14:2020/10/30[Date - Publication]) AND 2019/06/14:2020/10/30[Date - Publication] AND (("Clinical Trial"[Publication Type] OR "Randomized Controlled Trial"[Publication Type] OR "Random Allocation"[MeSH Terms] OR "Single Blind Method"[MeSH Terms] OR "Double Blind Method"[MeSH Terms] OR ("random*"[Title/Abstract] AND "trial*"[Title/Abstract]) OR ("random*"[Title/Abstract] AND "allocation*"[Title/Abstract]) OR ("blind*"[Title/Abstract] AND "method*"[Title/Abstract])) AND 2019/06/14:2020/10/23[Date - Publication])

Records Excluded (n=5)

-Substudy that did not meet entrance criteria

Records Excluded (n=100)

- Not a Cardiovascular Outcomes Trials (29)

- Not Randomized Control Trial (n=69)

- Not Human Trial (n=1)

- Not an SGLT-2i Trials (n=1)

Identification

Screening

Abstracts Screened

(n=109)

Records Identified through database searching

(n=109)

Papers Included in the Quantitative Synthesis. (n=12)

- 8 Main Study Reports (4 from initial review and 4 new from our review) and 4 secondary analysis in the original meta-analysis

Included

Eligibility

Full Text Articles Accessed

(n=9)

**Supplementary Figure 1:** Consort diagram for the incremental meta-analysis of randomized controlled trials of Sodium Glucose Co-transporter Two Inhibitors

**Supplementary Table 1** Risk of bias assessment

| **Study** | **Sequence**  **generation** | **Allocation**  **sequence**  **concealment** | **Blinding of**  **participants**  **and personnel** | **Blinding of**  **outcome**  **assessment** | **Incomplete**  **outcome data** | **Selective**  **outcome**  **reporting** |
| --- | --- | --- | --- | --- | --- | --- |
| **EMPA-REG Outcome** | Low risk | Low risk | Low risk | Low risk | Low risk | Low risk |
| **DECLARE-TIMI-58** | Low risk | Low risk | Low risk | Low risk | Low risk | Low risk |
| **CANVAS Program** | Low risk | Low risk | Low risk | Low risk | Low risk | Low risk |
| **VERTIS-CV** | Low risk | Low risk | Low risk | Low risk | Low risk | Low risk |
| **DAPA-HF** | Low risk | Low risk | Low risk | Low risk | Low risk | Low risk |
| **EMPEROR-REDUCED** | Low risk | Low risk | Low risk | Low risk | Low risk | Low risk |
| **CREDENCE** | Low risk | Low risk | Low risk | Low risk | Low risk | Low risk |
| **DAPA-CKD** | Low risk | Low risk | Low risk | Low risk | Low risk | Low risk |

**Supplementary Figure 1** Event rates (per 1000 patient years) for Heart Failure Hospitalizations (HHF, A) or the composite of Cardiovascular Death or HHF (B)

***
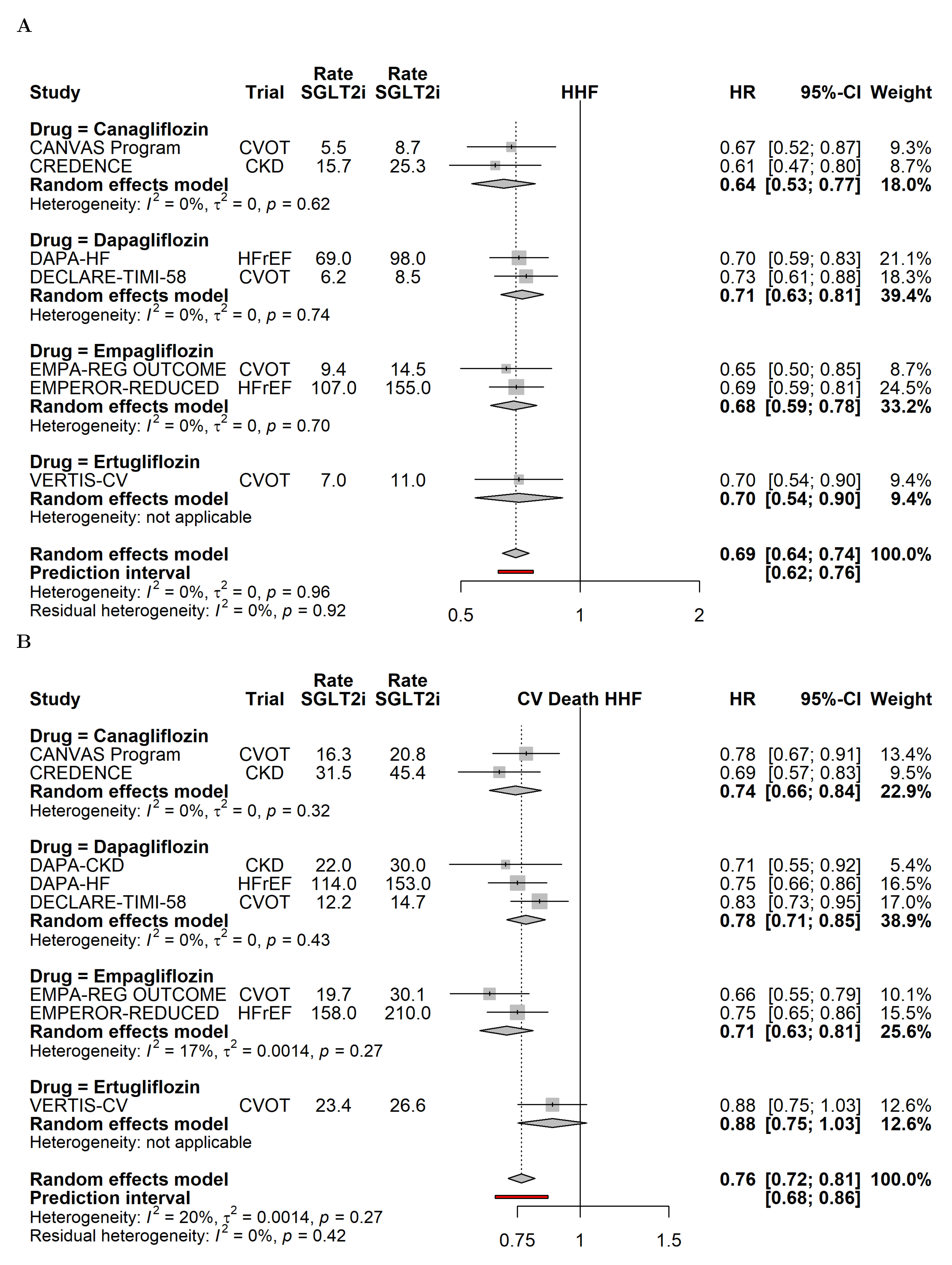
***

Random effects model synthesizes the effect across all studies, and the prediction interval gives the 95% range for the result of a future SGLT2i trial. CVOT: Cardiovascular Outcome Trial, HFrEF: Heart Failure with reduced Ejection Fraction, CKD: Chronic Kidney Disease, HR: Hazard Ratio

**Supplementary Figure 2** Event rates (per 1000 patient years) for non-fatal stroke in the SGLT2i trials
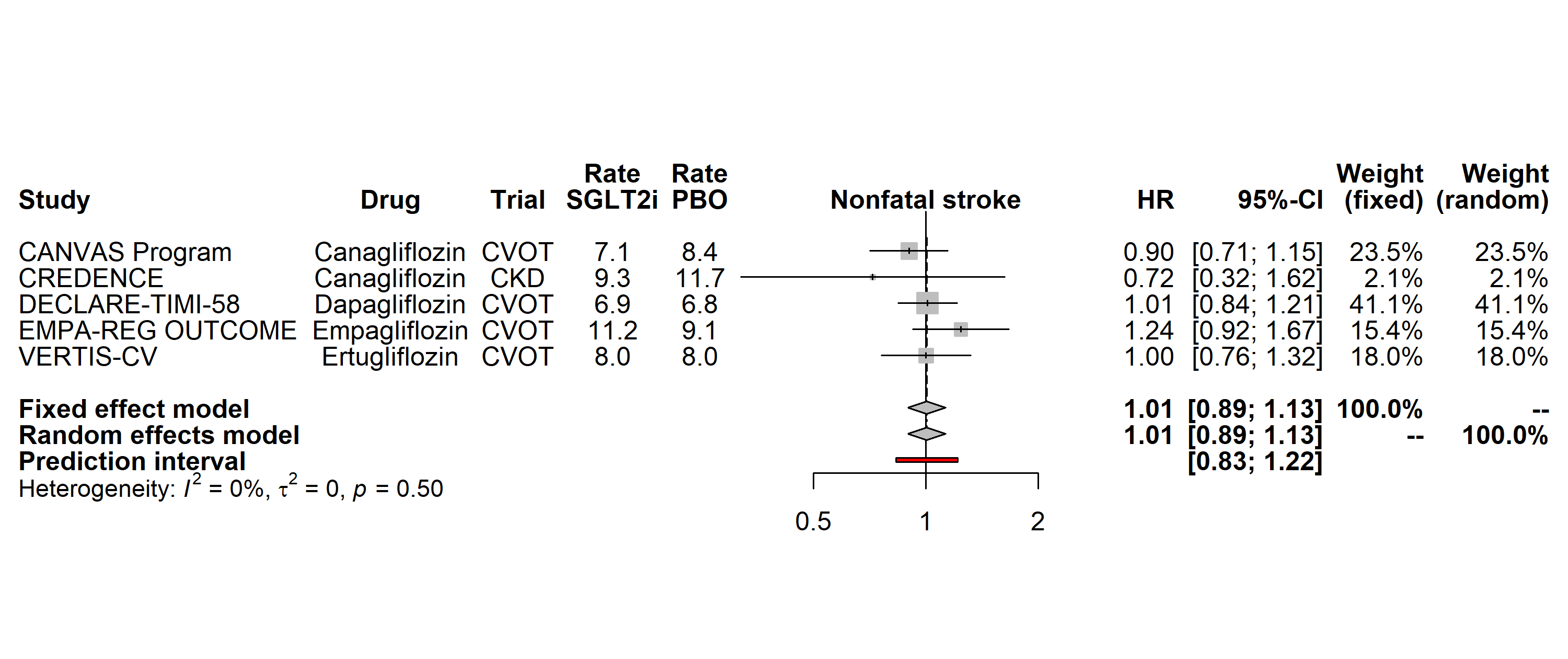


Effects of SGLT2i on nonfatal stroke . Random effects model synthesizes the effect across all studies, and the prediction interval gives the 95% range for the result of a future SGLT2i trial. CVOT: Cardiovascular Outcome Trial, CKD: Chronic Kidney Disease, Rate gives the event rates in each of the two arms of each trial.

**Supplementary Figure 3** Event rates (per 1000 patient years) for kidney outcomes in the SGLT2i trials
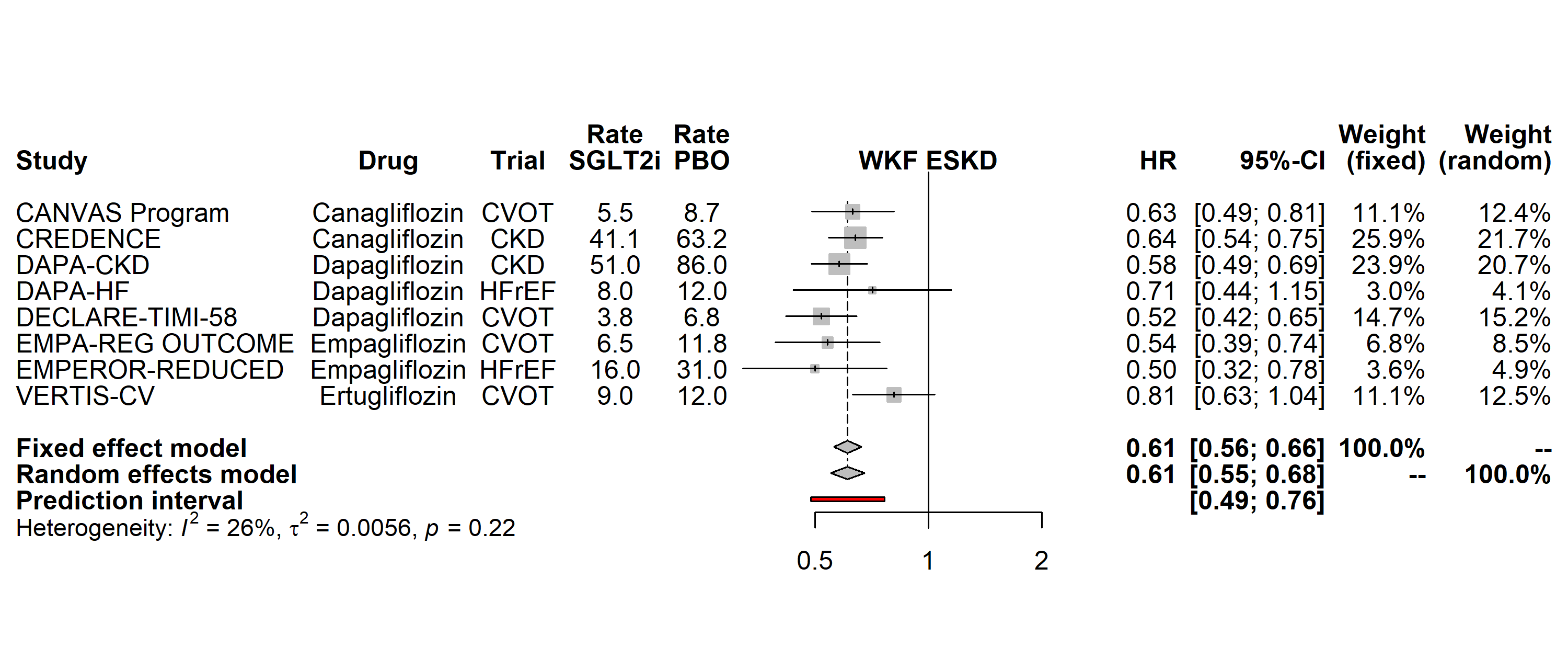


Effects of SGLT2i on the composite outcome of Worsening Kidney Function (WKD), i.e. decline of the estimated Glomerular Filtration Rate by at least 40%) or end stage kidney disease, i.e. need of dialysis or transplant. Random effects model synthesizes the effect across all studies, and the prediction interval gives the 95% range for the result of a future SGLT2i trial. CVOT: Cardiovascular Outcome Trial, HFrEF: Heart Failure with reduced Ejection Fraction, CKD: Chronic Kidney Disease, ESKD: End Stage Kidney Disease, HR: Hazard Ratio

**Citations of the 109 papers identified through the incremental meta-analysis process**

1: Kovil R, Saboo B, Shah K, Padhye D, Chudasama D, Raj V, Shaikh N. Single-pill

Combination of Empagliflozin and Linagliptin in Real World Indian Type 2

Diabetes Patient (GRID). J Assoc Physicians India. 2020 Oct;68(10):53-55. PMID:

32978926.

2: Heerspink HJL, Stefánsson BV, Correa-Rotter R, Chertow GM, Greene T, Hou FF,

Mann JFE, McMurray JJV, Lindberg M, Rossing P, Sjöström CD, Toto RD, Langkilde

AM, Wheeler DC; DAPA-CKD Trial Committees and Investigators. Dapagliflozin in

Patients with Chronic Kidney Disease. N Engl J Med. 2020 Oct

8;383(15):1436-1446. doi: 10.1056/NEJMoa2024816. Epub 2020 Sep 24. PMID:

32970396.

3: Filion KB, Lix LM, Yu OH, Dell'Aniello S, Douros A, Shah BR, St-Jean A,

Fisher A, Tremblay E, Bugden SC, Alessi-Severini S, Ronksley PE, Hu N, Dormuth

CR, Ernst P, Suissa S; Canadian Network for Observational Drug Effect Studies

(CNODES) Investigators. Sodium glucose cotransporter 2 inhibitors and risk of

major adverse cardiovascular events: multi-database retrospective cohort study.

BMJ. 2020 Sep 23;370:m3342. doi: 10.1136/bmj.m3342. PMID: 32967856.

4: Cannon CP, Pratley R, Dagogo-Jack S, Mancuso J, Huyck S, Masiukiewicz U,

Charbonnel B, Frederich R, Gallo S, Cosentino F, Shih WJ, Gantz I, Terra SG,

Cherney DZI, McGuire DK; VERTIS CV Investigators. Cardiovascular Outcomes with

Ertugliflozin in Type 2 Diabetes. N Engl J Med. 2020 Oct 8;383(15):1425-1435.

doi: 10.1056/NEJMoa2004967. Epub 2020 Sep 23. PMID: 32966714.

5: Zannad F, Ferreira JP, Pocock SJ, Anker SD, Butler J, Filippatos G,

Brueckmann M, Ofstad AP, Pfarr E, Jamal W, Packer M. SGLT2 inhibitors in

patients with heart failure with reduced ejection fraction: a meta-analysis of

the EMPEROR-Reduced and DAPA-HF trials. Lancet. 2020 Sep 19;396(10254):819-829.

doi: 10.1016/S0140-6736(20)31824-9. Epub 2020 Aug 30. PMID: 32877652.

6: Packer M, Anker SD, Butler J, Filippatos G, Pocock SJ, Carson P, Januzzi J,

Verma S, Tsutsui H, Brueckmann M, Jamal W, Kimura K, Schnee J, Zeller C, Cotton

D, Bocchi E, Böhm M, Choi DJ, Chopra V, Chuquiure E, Giannetti N, Janssens S,

Zhang J, Gonzalez Juanatey JR, Kaul S, Brunner-La Rocca HP, Merkely B, Nicholls

SJ, Perrone S, Pina I, Ponikowski P, Sattar N, Senni M, Seronde MF, Spinar J,

Squire I, Taddei S, Wanner C, Zannad F; EMPEROR-Reduced Trial Investigators.

Cardiovascular and Renal Outcomes with Empagliflozin in Heart Failure. N Engl J

Med. 2020 Oct 8;383(15):1413-1424. doi: 10.1056/NEJMoa2022190. Epub 2020 Aug 28.

PMID: 32865377.

7: Lo C, Toyama T, Oshima M, Jun M, Chin KL, Hawley CM, Zoungas S. Glucose-

lowering agents for treating pre-existing and new-onset diabetes in kidney

transplant recipients. Cochrane Database Syst Rev. 2020 Jul 30;8:CD009966. doi:

10.1002/14651858.CD009966.pub3. PMID: 32803882.

8: Zhuang Y, Song J, Ying M, Li M. Efficacy and safety of dapagliflozin plus

saxagliptin vs monotherapy as added to metformin in patients with type 2

diabetes: A meta-analysis. Medicine (Baltimore). 2020 Jul 24;99(30):e21409. doi:

10.1097/MD.0000000000021409. PMID: 32791755; PMCID: PMC7386974.

9: Khorlampenko AA, Karetnikova VN, Kochergina AM, Ignatova JS, Dyleva JA,

Gruzdeva OV, Barbarash OL. [Effect of empagliflosin on renal filtration in

patients with coronary heart disease undergoing percutaneous coronary

intervention]. Kardiologiia. 2020 Jul 7;60(6):825. Russian. doi:

10.18087/cardio.2020.6.n825. PMID: 32720618.

10: Cai T, Gao Y, Zhang L, Yang T, Chen Q. Effects of different dosages of

Sodium-Glucose Transporter 2 Inhibitors on lipid levels in patients with type 2

diabetes mellitus: A protocol for systematic review and meta-analysis. Medicine

(Baltimore). 2020 Jul 17;99(29):e20735. doi: 10.1097/MD.0000000000020735. PMID:

32702819; PMCID: PMC7373585.

11: Panevin TS, Eliseev MS, Shestakova MV, Nasonov EL. [Advantages of therapy

with sodium glucose cotransporter type 2 inhibitors in patients with type 2

diabetes mellitus in combination with hyperuricemia and gout]. Ter Arkh. 2020

Jun 5;92(5):110-118. Russian. doi: 10.26442/00403660.2020.05.000633. PMID:

32598783.

12: Neeland IJ, de Albuquerque Rocha N, Hughes C, Ayers CR, Malloy CR, Jin ES.

Effects of Empagliflozin Treatment on Glycerol-Derived Hepatic Gluconeogenesis

in Adults with Obesity: A Randomized Clinical Trial. Obesity (Silver Spring).

2020 Jul;28(7):1254-1262. doi: 10.1002/oby.22854. PMID: 32568464; PMCID:

PMC7316140.

13: Saleh S, Hanna G, El-Nabi SH, El-Domiaty H, Shabaan A, Fayez Ewida S.

Dapagliflozin, a sodium glucose cotransporter 2 inhibitors, protects

cardiovascular function in type-2 diabetic murine model. J Genet. 2020;99:46.

PMID: 32529989.

14: Arbel R, Aboalhasan E, Hammerman A, Azuri J. Sodium-Glucose Cotransporter 2

Inhibitors for Prevention of Heart Failure Events in Patients with Type 2

Diabetes Mellitus: A Cost Per Outcome Analysis. Clin Drug Investig. 2020

Jul;40(7):665-669. doi: 10.1007/s40261-020-00929-z. Erratum in: Clin Drug

Investig. 2020 Jun 6;: PMID: 32449083.

15: Lingvay I, Beetz N, Sennewald R, Schuler-Metz A, Bertulis J, Loley C, Lang

B, Lippert C, Lee J, Manning LS, Terada D. Triple fixed-dose combination

empagliflozin, linagliptin, and metformin for patients with type 2 diabetes.

Postgrad Med. 2020 May;132(4):337-345. doi: 10.1080/00325481.2020.1750228. Epub

2020 May 4. PMID: 32366156.

16: Iacobellis G, Gra-Menendez S. Effects of Dapagliflozin on Epicardial Fat

Thickness in Patients with Type 2 Diabetes and Obesity. Obesity (Silver Spring).

2020 Jun;28(6):1068-1074. doi: 10.1002/oby.22798. Epub 2020 Apr 30. PMID:

32352644.

17: Ghosh-Swaby OR, Goodman SG, Leiter LA, Cheng A, Connelly KA, Fitchett D,

Jüni P, Farkouh ME, Udell JA. Glucose-lowering drugs or strategies,

atherosclerotic cardiovascular events, and heart failure in people with or at

risk of type 2 diabetes: an updated systematic review and meta-analysis of

randomised cardiovascular outcome trials. Lancet Diabetes Endocrinol. 2020

May;8(5):418-435. doi: 10.1016/S2213-8587(20)30038-3. PMID: 32333878.

18: Miyashita S, Kuno T, Takagi H, Sugiyama T, Ando T, Valentin N, Shimada YJ,

Kodaira M, Numasawa Y, Kanei Y, Bangalore S. Risk of amputation associated with

sodium-glucose co-transporter 2 inhibitors: A meta-analysis of five randomized

controlled trials. Diabetes Res Clin Pract. 2020 May;163:108136. doi:

10.1016/j.diabres.2020.108136. Epub 2020 Apr 6. PMID: 32272190.

19: Merész G, Szabó S, Dóczy V, Hölgyesi Á, Szakács Z. A húgyúti fertőzések

relatív gyakorisága metforminnal és SGLT2-gátlóval kezelt 2-es típusú diabetes

mellitusban szenvedő betegekben. <i>Hálózati metaanalízis</i> [Relative

frequency of urinary tract infections in patients affected by diabetes mellitus

type 2 treated with metformin and SGLT2 inhibitor. <i>Network meta-

analysis</i>]. Orv Hetil. 2020 Mar;161(13):491-501. Hungarian. doi:

10.1556/650.2020.31690. PMID: 32202151.

20: Heymsfield SB, Raji A, Gallo S, Liu J, Pong A, Hannachi H, Terra SG.

Efficacy and Safety of Ertugliflozin in Patients with Overweight and Obesity

with Type 2 Diabetes Mellitus. Obesity (Silver Spring). 2020 Apr;28(4):724-732.

doi: 10.1002/oby.22748. PMID: 32202075; PMCID: PMC7217180.

21: Giugliano D, Longo M, Maiorino MI, Bellastella G, Chiodini P, Solerte SB,

Esposito K. Efficacy of SGLT-2 inhibitors in older adults with diabetes:

Systematic review with meta-analysis of cardiovascular outcome trials. Diabetes

Res Clin Pract. 2020 Apr;162:108114. doi: 10.1016/j.diabres.2020.108114. Epub

2020 Mar 9. PMID: 32165164.

22: Tsuda K. Letter by Tsuda Regarding Article, "Effect of Empagliflozin on Left

Ventricular Mass in Patients With Type 2 Diabetes Mellitus and Coronary Artery

Disease: The EMPA-HEART CardioLink-6 Randomized Clinical Trial". Circulation.

2020 Mar 3;141(9):e100-e101. doi: 10.1161/CIRCULATIONAHA.119.044977. Epub 2020

Mar 2. PMID: 32119586.

23: Tanaka M, Yamakage H, Inoue T, Odori S, Kusakabe T, Shimatsu A, Satoh-

Asahara N. Beneficial Effects of Ipragliflozin on the Renal Function and Serum

Uric Acid Levels in Japanese Patients with Type 2 Diabetes: A Randomized,

12-week, Open-label, Active-controlled Trial. Intern Med. 2020;59(5):601-609.

doi: 10.2169/internalmedicine.3473-19. Epub 2020 Mar 1. PMID: 32115517; PMCID:

PMC7086326.

24: Munk NE, Knudsen JS, Pottegård A, Witte DR, Thomsen RW. Differences Between

Randomized Clinical Trial Participants and Real-World Empagliflozin Users and

the Changes in Their Glycated Hemoglobin Levels. JAMA Netw Open. 2020 Feb

5;3(2):e1920949. doi: 10.1001/jamanetworkopen.2019.20949. PMID: 32031651.

25: Heerspink HJL, Stefansson BV, Chertow GM, Correa-Rotter R, Greene T, Hou FF,

Lindberg M, McMurray J, Rossing P, Toto R, Langkilde AM, Wheeler DC; DAPA-CKD

Investigators. Rationale and protocol of the Dapagliflozin And Prevention of

Adverse outcomes in Chronic Kidney Disease (DAPA-CKD) randomized controlled

trial. Nephrol Dial Transplant. 2020 Feb 1;35(2):274-282. doi:

10.1093/ndt/gfz290. PMID: 32030417; PMCID: PMC7005525.

26: Patoulias D, Stavropoulos K, Imprialos K, Athyros V, Doumas M, Karagiannis

A. Pharmacological Management of Cardiac Disease in Patients with Type 2

Diabetes: Insights into Clinical Practice. Curr Vasc Pharmacol.

2020;18(2):125-138. doi: 10.2174/1570161117666190426162746. PMID: 32013815.

27: Neuen BL, Jardine MJ, Perkovic V. Sodium-glucose cotransporter 2 inhibition:

which patient with chronic kidney disease should be treated in the future?

Nephrol Dial Transplant. 2020 Jan 1;35(Suppl 1):i48-i55. doi:

10.1093/ndt/gfz252. PMID: 32003833; PMCID: PMC6993192.

28: Zhang DP, Xu L, Wang LF, Wang HJ, Jiang F. Effects of antidiabetic drugs on

left ventricular function/dysfunction: a systematic review and network meta-

analysis. Cardiovasc Diabetol. 2020 Jan 22;19(1):10. doi:

10.1186/s12933-020-0987-x. PMID: 31969144; PMCID: PMC6977298.

29: Fuchigami A, Shigiyama F, Kitazawa T, Okada Y, Ichijo T, Higa M, Hiyoshi T,

Inoue I, Iso K, Yoshii H, Hirose T, Kumashiro N. Efficacy of dapagliflozin

versus sitagliptin on cardiometabolic risk factors in Japanese patients with

type 2 diabetes: a prospective, randomized study (DIVERSITY-CVR). Cardiovasc

Diabetol. 2020 Jan 7;19(1):1. doi: 10.1186/s12933-019-0977-z. PMID: 31910850;

PMCID: PMC6945792.

30: Tripolt NJ, Kolesnik E, Pferschy PN, Verheyen N, Ablasser K, Sailer S, Alber

H, Berger R, Kaulfersch C, Leitner K, Lichtenauer M, Mader A, Moertl D, Oulhaj

A, Reiter C, Rieder T, Saely CH, Siller-Matula J, Weidinger F, Zechner PM, von

Lewinski D, Sourij H; EMMY study group. Impact of EMpagliflozin on cardiac

function and biomarkers of heart failure in patients with acute MYocardial

infarction-The EMMY trial. Am Heart J. 2020 Mar;221:39-47. doi:

10.1016/j.ahj.2019.12.004. Epub 2019 Dec 12. PMID: 31901799.

31: Bjornstad P, Nelson RG, Pavkov ME. Do sodium-glucose cotransporter-2

inhibitors affect renal hemodynamics by different mechanisms in type 1 and type

2 diabetes? Kidney Int. 2020 Jan;97(1):31-33. doi: 10.1016/j.kint.2019.09.023.

PMID: 31901353; PMCID: PMC7075707.

32: Danjuma MI, Shokri SA, Saud AIYA, Elshafei MNA, Fatima H, Doi S, Bidmos MA.

Efficacy of sodium-glucose co-transporter 2 inhibitors in patients with type II

diabetes: A protocol for systematic review of randomised controlled clinical

trials utilising a generalised pairwise modelling methodology. Medicine

(Baltimore). 2019 Dec;98(51):e18198. doi: 10.1097/MD.0000000000018198. PMID:

31860967; PMCID: PMC6940159.

33: Butler J, Packer M, Greene SJ, Fiuzat M, Anker SD, Anstrom KJ, Carson PE,

Cooper LB, Fonarow GC, Hernandez AF, Januzzi JL Jr, Jessup M, Kalyani RR, Kaul

S, Kosiborod M, Lindenfeld J, McGuire DK, Sabatine MS, Solomon SD, Teerlink JR,

Vaduganathan M, Yancy CW, Stockbridge N, O'Connor CM. Heart Failure End Points

in Cardiovascular Outcome Trials of Sodium Glucose Cotransporter 2 Inhibitors in

Patients With Type 2 Diabetes Mellitus: A Critical Evaluation of Clinical and

Regulatory Issues. Circulation. 2019 Dec 17;140(25):2108-2118. doi:

10.1161/CIRCULATIONAHA.119.042155. Epub 2019 Dec 16. PMID: 31841369; PMCID:

PMC7027964.

34: Menne J, Dumann E, Haller H, Schmidt BMW. Acute kidney injury and adverse

renal events in patients receiving SGLT2-inhibitors: A systematic review and

meta-analysis. PLoS Med. 2019 Dec 9;16(12):e1002983. doi:

10.1371/journal.pmed.1002983. PMID: 31815931; PMCID: PMC6901179.

35: Bromage DI, Godec TR, Pujades-Rodriguez M, Gonzalez-Izquierdo A, Denaxas S,

Hemingway H, Yellon DM. Metformin use and cardiovascular outcomes after acute

myocardial infarction in patients with type 2 diabetes: a cohort study.

Cardiovasc Diabetol. 2019 Dec 9;18(1):168. doi: 10.1186/s12933-019-0972-4. PMID:

31815634; PMCID: PMC6900858.

36: Zou CY, Liu XK, Sang YQ, Wang B, Liang J. Effects of SGLT2 inhibitors on

cardiovascular outcomes and mortality in type 2 diabetes: A meta-analysis.

Medicine (Baltimore). 2019 Dec;98(49):e18245. doi: 10.1097/MD.0000000000018245.

PMID: 31804352; PMCID: PMC6919451.

37: Mirabelli M, Chiefari E, Caroleo P, Vero R, Brunetti FS, Corigliano DM,

Arcidiacono B, Foti DP, Puccio L, Brunetti A. Long-Term Effectiveness and Safety

of SGLT-2 Inhibitors in an Italian Cohort of Patients with Type 2 Diabetes

Mellitus. J Diabetes Res. 2019 Nov 4;2019:3971060. doi: 10.1155/2019/3971060.

PMID: 31781664; PMCID: PMC6875368.

38: Nassif ME, Kosiborod M. A Review of Cardiovascular Outcomes Trials of

Glucose-Lowering Therapies and Their Effects on Heart Failure Outcomes. Am J

Cardiol. 2019 Dec 15;124 Suppl 1:S12-S19. doi: 10.1016/j.amjcard.2019.10.025.

PMID: 31741435.

39: Patoulias D, Stavropoulos K, Imprialos K, Katsimardou A, Kalogirou MS,

Koutsampasopoulos K, Zografou I, Papadopoulos C, Karagiannis A, Doumas M.

Glycemic efficacy and safety of glucagon-like peptide-1 receptor agonist on top

of sodium-glucose co-transporter-2 inhibitor treatment compared to sodium-

glucose co-transporter-2 inhibitor alone: A systematic review and meta-analysis

of randomized controlled trials. Diabetes Res Clin Pract. 2019 Dec;158:107927.

doi: 10.1016/j.diabres.2019.107927. Epub 2019 Nov 13. PMID: 31733280.

40: Cannon CP, Perkovic V, Agarwal R, Baldassarre J, Bakris G, Charytan DM, de

Zeeuw D, Edwards R, Greene T, Heerspink HJL, Jardine MJ, Levin A, Li JW, Neal B,

Pollock C, Wheeler DC, Zhang H, Zinman B, Mahaffey KW. Evaluating the Effects of

Canagliflozin on Cardiovascular and Renal Events in Patients With Type 2

Diabetes Mellitus and Chronic Kidney Disease According to Baseline HbA1c,

Including Those With HbA1c <7%: Results From the CREDENCE Trial. Circulation.

2020 Feb 4;141(5):407-410. doi: 10.1161/CIRCULATIONAHA.119.044359. Epub 2019 Nov

11. PMID: 31707795.

41: Bhatt DL, Verma S, Braunwald E. The DAPA-HF Trial: A Momentous Victory in

the War against Heart Failure. Cell Metab. 2019 Nov 5;30(5):847-849. doi:

10.1016/j.cmet.2019.10.008. PMID: 31693879.

42: Tang Y, Sun Q, Bai XY, Zhou YF, Zhou QL, Zhang M. Effect of dapagliflozin on

obstructive sleep apnea in patients with type 2 diabetes: a preliminary study.

Nutr Diabetes. 2019 Nov 4;9(1):32. doi: 10.1038/s41387-019-0098-5. PMID:

31685792; PMCID: PMC6828696.

43: De Cosmo S, Viazzi F, Piscitelli P, Leoncini G, Mirijello A, Bonino B,

Pontremoli R. Impact of CVOTs in primary and secondary prevention of kidney

disease. Diabetes Res Clin Pract. 2019 Nov;157:107907. doi:

10.1016/j.diabres.2019.107907. Epub 2019 Oct 30. PMID: 31676332.

44: Chai S, Yu S, Yang Z, Wu S, Gao L, Wang H, Zhang Y, Zhan S, Ji L, Sun F.

Effect of incretin-based therapies on cancers of digestive system among 101 595

patients with type 2 diabetes mellitus: a systematic review and network meta-

analysis combining 84 trials with a median duration of 30 weeks. BMJ Open

Diabetes Res Care. 2019 Sep 20;7(1):e000728. doi: 10.1136/bmjdrc-2019-000728.

PMID: 31641525; PMCID: PMC6777405.

45: Monteiro P, Bergenstal RM, Toural E, Inzucchi SE, Zinman B, Hantel S, Kiš

SG, Kaspers S, George JT, Fitchett D. Efficacy and safety of empagliflozin in

older patients in the EMPA-REG OUTCOME® trial. Age Ageing. 2019 Nov

1;48(6):859-866. doi: 10.1093/ageing/afz096. PMID: 31579904.

46: Bhosle D, Quazi Z, Chavan S, Shaikh H. Efficacy and Safety of Canagliflozin

in Patients with Type II Diabetes Mellitus Inadequately Controlled on Triple

Drug Therapy. J Assoc Physicians India. 2019 Oct;67(10):36-38. PMID: 31571450.

47: Wang H, Yao G, Chen X, Ouyang J, Yang J. Ipragliflozin as an add-on therapy

in type 2 diabetes mellitus patients: An evidence-based pharmacoeconomics

evaluation. Diabetes Res Clin Pract. 2019 Nov;157:107867. doi:

10.1016/j.diabres.2019.107867. Epub 2019 Sep 27. PMID: 31568801.

48: Kaku K, Haneda M, Sakamaki H, Yasui A, Murata T, Ustyugova A, Chin R, Hirase

T, Shibahara T, Hayashi N, Kansal A, Kaspers S, Okamura T. Cost-effectiveness

Analysis of Empagliflozin in Japan Based on Results From the Asian subpopulation

in the EMPA-REG OUTCOME Trial. Clin Ther. 2019 Oct;41(10):2021-2040.e11. doi:

10.1016/j.clinthera.2019.07.016. Epub 2019 Sep 25. PMID: 31561882.

49: Lingvay I, Catarig AM, Frias JP, Kumar H, Lausvig NL, le Roux CW, Thielke D,

Viljoen A, McCrimmon RJ. Efficacy and safety of once-weekly semaglutide versus

daily canagliflozin as add-on to metformin in patients with type 2 diabetes

(SUSTAIN 8): a double-blind, phase 3b, randomised controlled trial. Lancet

Diabetes Endocrinol. 2019 Nov;7(11):834-844. doi: 10.1016/S2213-8587(19)30311-0.

Epub 2019 Sep 17. PMID: 31540867.

50: Scheen AJ. SGLT2 inhibitor or GLP-1 receptor agonist in type 2 diabetes?

Lancet Diabetes Endocrinol. 2019 Nov;7(11):818-820. doi:

10.1016/S2213-8587(19)30310-9. Epub 2019 Sep 17. PMID: 31540866.

51: McMurray JJV, Solomon SD, Inzucchi SE, Køber L, Kosiborod MN, Martinez FA,

Ponikowski P, Sabatine MS, Anand IS, Bělohlávek J, Böhm M, Chiang CE, Chopra VK,

de Boer RA, Desai AS, Diez M, Drozdz J, Dukát A, Ge J, Howlett JG, Katova T,

Kitakaze M, Ljungman CEA, Merkely B, Nicolau JC, O'Meara E, Petrie MC, Vinh PN,

Schou M, Tereshchenko S, Verma S, Held C, DeMets DL, Docherty KF, Jhund PS,

Bengtsson O, Sjöstrand M, Langkilde AM; DAPA-HF Trial Committees and

Investigators. Dapagliflozin in Patients with Heart Failure and Reduced Ejection

Fraction. N Engl J Med. 2019 Nov 21;381(21):1995-2008. doi:

10.1056/NEJMoa1911303. Epub 2019 Sep 19. PMID: 31535829.

52: Rodbard HW, Rosenstock J, Canani LH, Deerochanawong C, Gumprecht J, Lindberg

SØ, Lingvay I, Søndergaard AL, Treppendahl MB, Montanya E; PIONEER 2

Investigators. Oral Semaglutide Versus Empagliflozin in Patients With Type 2

Diabetes Uncontrolled on Metformin: The PIONEER 2 Trial. Diabetes Care. 2019

Dec;42(12):2272-2281. doi: 10.2337/dc19-0883. Epub 2019 Sep 17. PMID: 31530666.

53: Blonde L, Rosenstock J, Del Prato S, Henry R, Shehadeh N, Frias J,

Niemoeller E, Souhami E, Ji C, Aroda VR. Switching to iGlarLixi Versus

Continuing Daily or Weekly GLP-1 RA in Type 2 Diabetes Inadequately Controlled

by GLP-1 RA and Oral Antihyperglycemic Therapy: The LixiLan-G Randomized

Clinical Trial. Diabetes Care. 2019 Nov;42(11):2108-2116. doi:

10.2337/dc19-1357. Epub 2019 Sep 17. PMID: 31530665.

54: Neuen BL, Ohkuma T, Neal B, Matthews DR, de Zeeuw D, Mahaffey KW, Fulcher G,

Li Q, Jardine M, Oh R, Heerspink HL, Perkovic V. Effect of Canagliflozin on

Renal and Cardiovascular Outcomes across Different Levels of Albuminuria: Data

from the CANVAS Program. J Am Soc Nephrol. 2019 Nov;30(11):2229-2242. doi:

10.1681/ASN.2019010064. Epub 2019 Sep 17. PMID: 31530577; PMCID: PMC6830803.

55: Nassif ME, Windsor SL, Tang F, Khariton Y, Husain M, Inzucchi SE, McGuire

DK, Pitt B, Scirica BM, Austin B, Drazner MH, Fong MW, Givertz MM, Gordon RA,

Jermyn R, Katz SD, Lamba S, Lanfear DE, LaRue SJ, Lindenfeld J, Malone M,

Margulies K, Mentz RJ, Mutharasan RK, Pursley M, Umpierrez G, Kosiborod M.

Dapagliflozin Effects on Biomarkers, Symptoms, and Functional Status in Patients

With Heart Failure With Reduced Ejection Fraction: The DEFINE-HF Trial.

Circulation. 2019 Oct 29;140(18):1463-1476. doi:

10.1161/CIRCULATIONAHA.119.042929. Epub 2019 Sep 16. PMID: 31524498.

56: Hess DA, Terenzi DC, Trac JZ, Quan A, Mason T, Al-Omran M, Bhatt DL, Dhingra

N, Rotstein OD, Leiter LA, Zinman B, Sabongui S, Yan AT, Teoh H, Mazer CD,

Connelly KA, Verma S. SGLT2 Inhibition with Empagliflozin Increases Circulating

Provascular Progenitor Cells in People with Type 2 Diabetes Mellitus. Cell

Metab. 2019 Oct 1;30(4):609-613. doi: 10.1016/j.cmet.2019.08.015. Epub 2019 Aug

30. PMID: 31477497.

57: Fei Y, Tsoi MF, Cheung BMY. Cardiovascular outcomes in trials of new

antidiabetic drug classes: a network meta-analysis. Cardiovasc Diabetol. 2019

Aug 28;18(1):112. doi: 10.1186/s12933-019-0916-z. PMID: 31462224; PMCID:

PMC6714383.

58: Kinguchi S, Wakui H, Ito Y, Kondo Y, Azushima K, Osada U, Yamakawa T,

Iwamoto T, Yutoh J, Misumi T, Aoki K, Yasuda G, Yoshii T, Yamada T, Ono S,

Shibasaki-Kurita T, Hosokawa S, Orime K, Hanaoka M, Sasaki H, Inazumi K, Yamada

T, Kobayashi R, Ohki K, Haruhara K, Kobayashi Y, Yamanaka T, Terauchi Y, Tamura

K. Improved home BP profile with dapagliflozin is associated with amelioration

of albuminuria in Japanese patients with diabetic nephropathy: the Yokohama add-

on inhibitory efficacy of dapagliflozin on albuminuria in Japanese patients with

type 2 diabetes study (Y-AIDA study). Cardiovasc Diabetol. 2019 Aug

27;18(1):110. doi: 10.1186/s12933-019-0912-3. PMID: 31455298; PMCID: PMC6710883.

59: Nishimura R, Tanaka Y, Koiwai K, Ishida K, Salsali A, Kaspers S, Kohler S,

Lund SS. Effect of Empagliflozin on Free Fatty Acids and Ketone Bodies in

Japanese Patients with Type 2 Diabetes Mellitus: A Randomized Controlled Trial.

Adv Ther. 2019 Oct;36(10):2769-2782. doi: 10.1007/s12325-019-01045-x. Epub 2019

Aug 23. PMID: 31444706.

60: Buch A, Eldor R, Kis O, Keinan-Boker L, Dunsky A, Rubin A, Lopez A, Sofer Y,

Osher E, Marcus Y, Stern N. The effect of circuit resistance training,

empagliflozin or "vegeterranean diet" on physical and metabolic function in

older subjects with type 2 diabetes: a study protocol for a randomized control

trial (CEV-65 trial). BMC Geriatr. 2019 Aug 22;19(1):228. doi:

10.1186/s12877-019-1219-7. PMID: 31438863; PMCID: PMC6704502.

61: Verma S, Mazer CD, Yan AT, Mason T, Garg V, Teoh H, Zuo F, Quan A, Farkouh

ME, Fitchett DH, Goodman SG, Goldenberg RM, Al-Omran M, Gilbert RE, Bhatt DL,

Leiter LA, Jüni P, Zinman B, Connelly KA. Effect of Empagliflozin on Left

Ventricular Mass in Patients With Type 2 Diabetes Mellitus and Coronary Artery

Disease: The EMPA-HEART CardioLink-6 Randomized Clinical Trial. Circulation.

2019 Nov 19;140(21):1693-1702. doi: 10.1161/CIRCULATIONAHA.119.042375. Epub 2019

Aug 22. PMID: 31434508.

62: Abdelgadir E, Rashid F, Bashier A, Al Saeed M, Khalifa A, Alawadi F,

Hassanein M. Use of flash glucose monitoring system in assessing safety of the

SGLT2 inhibitors during Ramadan fasting in high risk insulin treated patients

with type 2 diabetes. Diabetes Metab Syndr. 2019 Sep-Oct;13(5):2927-2932. doi:

10.1016/j.dsx.2019.07.055. Epub 2019 Jul 30. PMID: 31425958.

63: Bonora BM, Vigili de Kreutzenberg S, Avogaro A, Fadini GP. Effects of the

SGLT2 inhibitor dapagliflozin on cardiac function evaluated by impedance

cardiography in patients with type 2 diabetes. Secondary analysis of a

randomized placebo-controlled trial. Cardiovasc Diabetol. 2019 Aug 14;18(1):106.

doi: 10.1186/s12933-019-0910-5. PMID: 31412874; PMCID: PMC6694690.

64: Wu P, Wen W, Li J, Xu J, Zhao M, Chen H, Sun J. Systematic Review and Meta-

Analysis of Randomized Controlled Trials on the Effect of SGLT2 Inhibitor on

Blood Leptin and Adiponectin Level in Patients with Type 2 Diabetes. Horm Metab

Res. 2019 Aug;51(8):487-494. doi: 10.1055/a-0958-2441. Epub 2019 Aug 13. PMID:

31408894.

65: O'Keefe JH, Nassif ME, Magwire ML, O'Keefe EL, Lavie CJ. The elephant in the

room: Why cardiologists should stop ignoring type 2 diabetes. Prog Cardiovasc

Dis. 2019 Jul-Aug;62(4):364-369. doi: 10.1016/j.pcad.2019.08.001. Epub 2019 Aug

10. PMID: 31408637.

66: Dekkers CCJ, Sjöström CD, Greasley PJ, Cain V, Boulton DW, Heerspink HJL.

Effects of the sodium-glucose co-transporter-2 inhibitor dapagliflozin on

estimated plasma volume in patients with type 2 diabetes. Diabetes Obes Metab.

2019 Dec;21(12):2667-2673. doi: 10.1111/dom.13855. Epub 2019 Sep 17. PMID:

31407856; PMCID: PMC6899523.

67: Lan NSR, Fegan PG, Yeap BB, Dwivedi G. The effects of sodium-glucose

cotransporter 2 inhibitors on left ventricular function: current evidence and

future directions. ESC Heart Fail. 2019 Oct;6(5):927-935. doi:

10.1002/ehf2.12505. Epub 2019 Aug 10. PMID: 31400090; PMCID: PMC6816235.

68: Zhou Z, Jardine M, Perkovic V, Matthews DR, Mahaffey KW, de Zeeuw D, Fulcher

G, Desai M, Oh R, Simpson R, Watts NB, Neal B. Canagliflozin and fracture risk

in individuals with type 2 diabetes: results from the CANVAS Program.

Diabetologia. 2019 Oct;62(10):1854-1867. doi: 10.1007/s00125-019-4955-5. Epub

2019 Aug 10. PMID: 31399845; PMCID: PMC6731200.

69: Bailey CJ, Del Prato S, Wei C, Reyner D, Saraiva G. Durability of glycaemic

control with dapagliflozin, an SGLT2 inhibitor, compared with saxagliptin, a

DPP4 inhibitor, in patients with inadequately controlled type 2 diabetes.

Diabetes Obes Metab. 2019 Nov;21(11):2564-2569. doi: 10.1111/dom.13841. Epub

2019 Aug 26. PMID: 31364269; PMCID: PMC6851837.

70: Feng M, Lv H, Xu X, Wang J, Lyu W, Fu S. Efficacy and safety of

dapagliflozin as monotherapy in patients with type 2 diabetes mellitus: A meta-

analysis of randomized controlled trials. Medicine (Baltimore). 2019

Jul;98(30):e16575. doi: 10.1097/MD.0000000000016575. PMID: 31348290; PMCID:

PMC6709066.

71: Kanters S, Wilkinson L, Vrazic H, Sharma R, Lopes S, Popoff E, Druyts E.

Comparative efficacy of once-weekly semaglutide versus SGLT-2 inhibitors in

patients inadequately controlled with one to two oral antidiabetic drugs: a

systematic literature review and network meta-analysis. BMJ Open. 2019 Jul

23;9(7):e023458. doi: 10.1136/bmjopen-2018-023458. PMID: 31340953; PMCID:

PMC6661926.

72: Halvorsen YC, Walford GA, Massaro J, Aftring RP, Freeman MW. A 96-week,

multinational, randomized, double-blind, parallel-group, clinical trial

evaluating the safety and effectiveness of bexagliflozin as a monotherapy for

adults with type 2 diabetes. Diabetes Obes Metab. 2019 Nov;21(11):2496-2504.

doi: 10.1111/dom.13833. Epub 2019 Aug 8. PMID: 31297965.

73: Mahaffey KW, Jardine MJ, Bompoint S, Cannon CP, Neal B, Heerspink HJL,

Charytan DM, Edwards R, Agarwal R, Bakris G, Bull S, Capuano G, de Zeeuw D,

Greene T, Levin A, Pollock C, Sun T, Wheeler DC, Yavin Y, Zhang H, Zinman B,

Rosenthal N, Brenner BM, Perkovic V. Canagliflozin and Cardiovascular and Renal

Outcomes in Type 2 Diabetes Mellitus and Chronic Kidney Disease in Primary and

Secondary Cardiovascular Prevention Groups. Circulation. 2019 Aug

27;140(9):739-750. doi: 10.1161/CIRCULATIONAHA.119.042007. Epub 2019 Jul 11.

PMID: 31291786; PMCID: PMC6727954.

74: Shin JI. Second-line Glucose-Lowering Therapy in Type 2 Diabetes Mellitus.

Curr Diab Rep. 2019 Jul 8;19(8):54. doi: 10.1007/s11892-019-1171-0. PMID:

31286271.

75: Jingfan Z, Ling L, Cong L, Ping L, Yu C. Efficacy and safety of sodium-

glucose cotransporter-2 inhibitors in type 2 diabetes mellitus with inadequate

glycemic control on metformin: a meta-analysis. Arch Endocrinol Metab. 2019 Jun

27;63(5):478-486. doi: 10.20945/2359-3997000000146. PMID: 31271575.

76: Mulder S, Heerspink HJL, Darshi M, Kim JJ, Laverman GD, Sharma K, Pena MJ.

Effects of dapagliflozin on urinary metabolites in people with type 2 diabetes.

Diabetes Obes Metab. 2019 Nov;21(11):2422-2428. doi: 10.1111/dom.13823. Epub

2019 Jul 18. PMID: 31264758.

77: Jensen J, Omar M, Kistorp C, Poulsen MK, Tuxen C, Gustafsson I, Køber L,

Gustafsson F, Fosbøl E, Bruun NE, Videbæk L, Frederiksen PH, Møller JE, Schou M.

Empagliflozin in heart failure patients with reduced ejection fraction: a

randomized clinical trial (Empire HF). Trials. 2019 Jun 21;20(1):374. doi:

10.1186/s13063-019-3474-5. PMID: 31227014; PMCID: PMC6588901.

78: Abraham WT, Ponikowski P, Brueckmann M, Zeller C, Macesic H, Peil B, Brun M,

Ustyugova A, Jamal W, Salsali A, Lindenfeld J, Anker SD; EMPERIAL Investigators

and National Coordinators. Rationale and design of the EMPERIAL-Preserved and

EMPERIAL-Reduced trials of empagliflozin in patients with chronic heart failure.

Eur J Heart Fail. 2019 Jul;21(7):932-942. doi: 10.1002/ejhf.1486. Epub 2019 Jun

19. PMID: 31218819; PMCID: PMC6774309.

79: Kurozumi A, Okada Y, Shimokawa M, Goshima Y, Otsuka T, Narisawa M, Torimoto

K, Tanaka Y. Efficacy and Safety of Tofogliflozin on 24-h Glucose Profile Based

on Continuous Glucose Monitoring: Crossover Study of Sodium-Glucose

Cotransporter 2 Inhibitor. Diabetes Technol Ther. 2019 Jul;21(7):385-392. doi:

10.1089/dia.2019.0099. Epub 2019 Jun 17. PMID: 31210529.

80: Mosenzon O, Wiviott SD, Cahn A, Rozenberg A, Yanuv I, Goodrich EL, Murphy

SA, Heerspink HJL, Zelniker TA, Dwyer JP, Bhatt DL, Leiter LA, McGuire DK,

Wilding JPH, Kato ET, Gause-Nilsson IAM, Fredriksson M, Johansson PA, Langkilde

AM, Sabatine MS, Raz I. Effects of dapagliflozin on development and progression

of kidney disease in patients with type 2 diabetes: an analysis from the

DECLARE-TIMI 58 randomised trial. Lancet Diabetes Endocrinol. 2019

Aug;7(8):606-617. doi: 10.1016/S2213-8587(19)30180-9. Epub 2019 Jun 10. Erratum

in: Lancet Diabetes Endocrinol. 2019 Aug;7(8):e20. PMID: 31196815.

81: Pratley R, Amod A, Hoff ST, Kadowaki T, Lingvay I, Nauck M, Pedersen KB,

Saugstrup T, Meier JJ; PIONEER 4 investigators. Oral semaglutide versus

subcutaneous liraglutide and placebo in type 2 diabetes (PIONEER 4): a

randomised, double-blind, phase 3a trial. Lancet. 2019 Jul 6;394(10192):39-50.

doi: 10.1016/S0140-6736(19)31271-1. Epub 2019 Jun 8. Erratum in: Lancet. 2019

Jul 6;394(10192):e1. PMID: 31186120.

82: Mishriky BM, Powell JR, Wittwer JA, Chu JX, Sewell KA, Wu Q, Cummings DM. Do

GLP-1RAs and SGLT-2is reduce cardiovascular events in black patients with type 2

diabetes? A systematic review and meta-analysis. Diabetes Obes Metab. 2019

Oct;21(10):2274-2283. doi: 10.1111/dom.13805. Epub 2019 Jun 30. PMID: 31168889.

83: Vilsbøll T, Ekholm E, Johnsson E, Dronamraju N, Jabbour S, Lind M.

Dapagliflozin Plus Saxagliptin Add-on Therapy Compared With Insulin in Patients

With Type 2 Diabetes Poorly Controlled by Metformin With or Without Sulfonylurea

Therapy: A Randomized Clinical Trial. Diabetes Care. 2019 Aug;42(8):1464-1472.

doi: 10.2337/dc18-1988. Epub 2019 Jun 4. PMID: 31167892.

84: Solini A, Seghieri M, Giannini L, Biancalana E, Parolini F, Rossi C, Dardano

A, Taddei S, Ghiadoni L, Bruno RM. The Effects of Dapagliflozin on Systemic and

Renal Vascular Function Display an Epigenetic Signature. J Clin Endocrinol

Metab. 2019 Oct 1;104(10):4253-4263. doi: 10.1210/jc.2019-00706. PMID: 31162549.

85: Halvorsen YD, Lock JP, Zhou W, Zhu F, Freeman MW. A 24-week, randomized,

double-blind, active-controlled clinical trial comparing bexagliflozin with

sitagliptin as an adjunct to metformin for the treatment of type 2 diabetes in

adults. Diabetes Obes Metab. 2019 Oct;21(10):2248-2256. doi: 10.1111/dom.13801.

Epub 2019 Jun 30. PMID: 31161692.

86: Dicembrini I, Tomberli B, Nreu B, Baldereschi GI, Fanelli F, Mannucci E,

Monami M. Peripheral artery disease and amputations with Sodium-Glucose co-

Transporter-2 (SGLT-2) inhibitors: A meta-analysis of randomized controlled

trials. Diabetes Res Clin Pract. 2019 Jul;153:138-144. doi:

10.1016/j.diabres.2019.05.028. Epub 2019 May 28. PMID: 31150722.

87: Rosenstock J, Perl S, Johnsson E, García-Sánchez R, Jacob S. Triple therapy

with low-dose dapagliflozin plus saxagliptin versus dual therapy with each

monocomponent, all added to metformin, in uncontrolled type 2 diabetes. Diabetes

Obes Metab. 2019 Sep;21(9):2152-2162. doi: 10.1111/dom.13795. Epub 2019 Jun 24.

PMID: 31144431; PMCID: PMC6771748.

88: Mayer GJ, Wanner C, Weir MR, Inzucchi SE, Koitka-Weber A, Hantel S, von

Eynatten M, Zinman B, Cherney DZI. Analysis from the EMPA-REG

OUTCOME<sup>®</sup> trial indicates empagliflozin may assist in

preventing the progression of chronic kidney disease in patients with type 2

diabetes irrespective of medications that alter intrarenal hemodynamics. Kidney

Int. 2019 Aug;96(2):489-504. doi: 10.1016/j.kint.2019.02.033. Epub 2019 Mar 21.

PMID: 31142441.

89: Hidayat K, Du X, Shi BM. Risk of fracture with dipeptidyl peptidase-4

inhibitors, glucagon-like peptide-1 receptor agonists, or sodium-glucose

cotransporter-2 inhibitors in real-world use: systematic review and meta-

analysis of observational studies. Osteoporos Int. 2019 Oct;30(10):1923-1940.

doi: 10.1007/s00198-019-04968-x. Epub 2019 May 27. PMID: 31134305.

90: Weir MR. Renal effects of sodium-glucose cotransporter-2 inhibitors in

patients with type 2 diabetes and renal impairment. Postgrad Med. 2019

Aug;131(6):367-375. doi: 10.1080/00325481.2019.1624582. Epub 2019 Jun 5. PMID:

31132013.

91: Allegretti AS, Zhang W, Zhou W, Thurber TK, Rigby SP, Bowman-Stroud C,

Trescoli C, Serusclat P, Freeman MW, Halvorsen YC. Safety and Effectiveness of

Bexagliflozin in Patients With Type 2 Diabetes Mellitus and Stage 3a/3b CKD. Am

J Kidney Dis. 2019 Sep;74(3):328-337. doi: 10.1053/j.ajkd.2019.03.417. Epub 2019

May 14. PMID: 31101403.

92: Liu J, Tarasenko L, Terra SG, Huyck S, Wu L, Pong A, Calle RA, Gallo S,

Darekar A, Mancuso JP. Efficacy of ertugliflozin in monotherapy or combination

therapy in patients with type 2 diabetes: A pooled analysis of placebo-

controlled studies. Diab Vasc Dis Res. 2019 Sep;16(5):415-423. doi:

10.1177/1479164119842513. Epub 2019 May 13. PMID: 31081371.

93: Torimoto K, Okada Y, Goshima Y, Tokutsu A, Sato Y, Tanaka Y. Addition of

canagliflozin to insulin improves glycaemic control and reduces insulin dose in

patients with type 2 diabetes mellitus: A randomized controlled trial. Diabetes

Obes Metab. 2019 Sep;21(9):2174-2179. doi: 10.1111/dom.13770. Epub 2019 Jun 11.

PMID: 31074205.

94: Gilbert RE, Thorpe KE. Acute kidney injury with sodium-glucose co-

transporter-2 inhibitors: A meta-analysis of cardiovascular outcome trials.

Diabetes Obes Metab. 2019 Aug;21(8):1996-2000. doi: 10.1111/dom.13754. Epub 2019

May 24. PMID: 31050116.

95: Terauchi Y, Fujiwara H, Kurihara Y, Suganami H, Tamura M, Senda M, Gunji R,

Kaku K. Long-term safety and efficacy of the sodium-glucose cotransporter 2

inhibitor, tofogliflozin, added on glucagon-like peptide-1 receptor agonist in

Japanese patients with type 2 diabetes mellitus: A 52-week open-label,

multicenter, post-marketing clinical study. J Diabetes Investig. 2019

Nov;10(6):1518-1526. doi: 10.1111/jdi.13066. Epub 2019 May 28. PMID: 31033218;

PMCID: PMC6825952.

96: Giugliano D, Maiorino MI, Longo M, Bellastella G, Chiodini P, Esposito K.

Type 2 diabetes and risk of heart failure: a systematic review and meta-analysis

from cardiovascular outcome trials. Endocrine. 2019 Jul;65(1):15-24. doi:

10.1007/s12020-019-01931-y. Epub 2019 Apr 26. PMID: 31028667.

97: Saito M, Kaibara A, Kadokura T, Toyoshima J, Yoshida S, Kazuta K, Ueyama E.

Pharmacokinetic and pharmacodynamic modelling for renal function dependent

urinary glucose excretion effect of ipragliflozin, a selective sodium-glucose

cotransporter 2 inhibitor, both in healthy subjects and patients with type 2

diabetes mellitus. Br J Clin Pharmacol. 2019 Aug;85(8):1808-1819. doi:

10.1111/bcp.13972. Epub 2019 Jun 20. PMID: 31026084; PMCID: PMC6624389.

98: Li X, Li T, Cheng Y, Lu Y, Xue M, Xu L, Liu X, Yu X, Sun B, Chen L. Effects

of SGLT2 inhibitors on fractures and bone mineral density in type 2 diabetes: An

updated meta-analysis. Diabetes Metab Res Rev. 2019 Oct;35(7):e3170. doi:

10.1002/dmrr.3170. Epub 2019 May 15. PMID: 30983141.

99: Athyros VG, Polyzos SA, Kountouras J, Katsiki N, Anagnostis P, Doumas M,

Mantzoros CS. Non-Alcoholic Fatty Liver Disease Treatment in Patients with Type

2 Diabetes Mellitus; New Kids on the Block. Curr Vasc Pharmacol.

2020;18(2):172-181. doi: 10.2174/1570161117666190405164313. PMID: 30961499.

100: Vinke JSJ, Heerspink HJL, de Borst MH. Effects of sodium glucose

cotransporter 2 inhibitors on mineral metabolism in type 2 diabetes mellitus.

Curr Opin Nephrol Hypertens. 2019 Jul;28(4):321-327. doi:

10.1097/MNH.0000000000000505. PMID: 30958403; PMCID: PMC6587226.

101: Hupfeld C, Mudaliar S. Navigating the "MACE" in Cardiovascular Outcomes

Trials and decoding the relevance of Atherosclerotic Cardiovascular Disease

benefits versus Heart Failure benefits. Diabetes Obes Metab. 2019

Aug;21(8):1780-1789. doi: 10.1111/dom.13740. Epub 2019 Apr 29. PMID: 30957945.

102: Giugliano D, Chiodini P, Maiorino MI, Bellastella G, Esposito K.

Cardiovascular outcome trials and major cardiovascular events: does glucose

matter? A systematic review with meta-analysis. J Endocrinol Invest. 2019

Oct;42(10):1165-1169. doi: 10.1007/s40618-019-01047-0. Epub 2019 Apr 6. PMID:

30955180.

103: Nunoi K, Sato Y, Kaku K, Yoshida A, Suganami H. Renal effects of a sodium-

glucose cotransporter 2 inhibitor, tofogliflozin, in relation to sodium intake

and glycaemic status. Diabetes Obes Metab. 2019 Jul;21(7):1715-1724. doi:

10.1111/dom.13731. Epub 2019 May 6. PMID: 30945431; PMCID: PMC6619387.

104: Yoshida A, Matsubayashi Y, Nojima T, Suganami H, Abe T, Ishizawa M,

Fujihara K, Tanaka S, Kaku K, Sone H. Attenuation of Weight Loss Through

Improved Antilipolytic Effect in Adipose Tissue Via the SGLT2 Inhibitor

Tofogliflozin. J Clin Endocrinol Metab. 2019 Sep 1;104(9):3647-3660. doi:

10.1210/jc.2018-02254. PMID: 30811541.

105: Hollander P, Hill J, Johnson J, Wei Jiang Z, Golm G, Huyck S, Terra SG,

Mancuso JP, Engel SS, Lauring B, Liu J. Results of VERTIS SU extension study:

safety and efficacy of ertugliflozin treatment over 104 weeks compared to

glimepiride in patients with type 2 diabetes mellitus inadequately controlled on

metformin. Curr Med Res Opin. 2019 Aug;35(8):1335-1343. doi:

10.1080/03007995.2019.1583450. Epub 2019 Mar 25. PMID: 30760125.

106: Cheng L, Li YY, Hu W, Bai F, Hao HR, Yu WN, Mao XM. Risk of bone fracture

associated with sodium-glucose cotransporter-2 inhibitor treatment: A meta-

analysis of randomized controlled trials. Diabetes Metab. 2019

Oct;45(5):436-445. doi: 10.1016/j.diabet.2019.01.010. Epub 2019 Feb 6. PMID:

30738154.

107: Kanazawa K, Uchino H, Shigiyama F, Igarashi H, Ikehara K, Yoshikawa F, Usui

S, Miyagi M, Yoshino H, Ando Y, Kumashiro N, Hirose T. Sustained fasting glucose

oxidation and postprandial lipid oxidation associated with reduced insulin dose

in type 2 diabetes with sodium-glucose cotransporter 2 inhibitor: A randomized,

open-label, prospective study. J Diabetes Investig. 2019 Jul;10(4):1022-1031.

doi: 10.1111/jdi.12994. Epub 2019 Feb 19. PMID: 30582774; PMCID: PMC6626995.

108: Inoue H, Morino K, Ugi S, Tanaka-Mizuno S, Fuse K, Miyazawa I, Kondo K,

Sato D, Ohashi N, Ida S, Sekine O, Yoshimura M, Murata K, Miura K, Arima H,

Maegawa H; SUMS-ADDIT-1 Research group. Ipragliflozin, a sodium-glucose

cotransporter 2 inhibitor, reduces bodyweight and fat mass, but not muscle mass,

in Japanese type 2 diabetes patients treated with insulin: A randomized clinical

trial. J Diabetes Investig. 2019 Jul;10(4):1012-1021. doi: 10.1111/jdi.12985.

Epub 2019 Jan 21. PMID: 30536746; PMCID: PMC6626939.

109: Inoue M, Hayashi A, Taguchi T, Arai R, Sasaki S, Takano K, Inoue Y,

Shichiri M. Effects of canagliflozin on body composition and hepatic fat content

in type 2 diabetes patients with non-alcoholic fatty liver disease. J Diabetes

Investig. 2019 Jul;10(4):1004-1011. doi: 10.1111/jdi.12980. Epub 2019 Jan 4.

PMID: 30461221; PMCID: PMC6626966.
